# “One less thing to worry about”: Barriers and facilitators of PrEP uptake among MSM in Karachi, Pakistan: A qualitative study

**DOI:** 10.1371/journal.pgph.0007151

**Published:** 2026-08-28

**Authors:** Batool Fatima, Sarmad Soomar, Preet Katyara, Shariqa Batool, Syed Faisal Mahmood

**Affiliations:** 1 Department of Community Health Sciences, Aga Khan University, Karachi, Pakistan; 2 School of Nursing and Midwifery, Aga Khan University, Karachi, Pakistan; 3 Internal Medicine, Henry Ford Hospital, Detroit, Michigan, United States of America; 4 Department of Medicine, Aga Khan University, Karachi, Pakistan; New York University, UNITED STATES OF AMERICA

## Abstract

In Pakistan, MSM represent one of the key populations at risk of HIV, with rising rates of HIV compared to the general population and other key populations. Pre-exposure prophylaxis (PrEP) was rolled out in Pakistan in two of the most populated provinces, Sindh and Punjab, targeting key populations including MSM. However, only 23% of the high-risk population in Sindh and only 9% in Punjab used PrEP. A qualitative study with a descriptive exploratory design was conducted to explore the barriers, willingness, and preferred ways of using PrEP among Men who have Sex with Men (MSM) in Karachi, Sindh. In-depth interviews were conducted with six MSM in the outpatient setting of a tertiary care hospital in Karachi, where PrEP is prescribed and available for use in the MSM population. Participants reported lack of awareness, fear of stigma and discrimination, concerns about privacy and confidentiality, and financial and logistical challenges as barriers to their willingness and uptake of PrEP. Facilitators included awareness, accessibility, and availability of PrEP; perceived benefits of PrEP; affordability; social support; and experiences of professionalism and non-judgmental care in PrEP services. Participants recommended raising awareness at MSM social gatherings or on MSM dating applications such as Grindr, ensuring privacy, anonymity, and comfort by providing online consulting services and male care providers, the possibility of home testing and delivery of PrEP medication, and the need for emotional support and validation of concerns when needed. They also expressed a desire for PrEP with a longer shelf life or long-acting injections. This study provides insights into the enabling factors and roadblocks in the uptake of PrEP among MSM and provides an opportunity to refine Human Immunodeficiency Virus (HIV) prevention services.

## Introduction

With an estimated global prevalence of 37 million in 2018 and approximately 1.7 million new infections every year, the Human Immunodeficiency Virus (HIV) epidemic continues to be a significant public health concern [1]. With about 250,000 people living with HIV, Pakistan's current HIV prevalence among the general population is estimated to be around 0.1%, concentrated in key populations, including intravenous (IV) drug abusers, men who have sex with men (MSM), female sex workers, and transgender people [2].

Men who have sex with men (MSM) in Pakistan face significant barriers to accessing HIV prevention services, owing to societal stigma and conservative cultural norms. This population remains highly vulnerable to HIV; however, structural, behavioral, and biomedical challenges hinder effective prevention strategies. Studies indicate that, among HIV-positive MSM in Pakistan, 58% are transgender, 39% have no formal education, and 76% lack financial support from their families, underscoring the socioeconomic vulnerabilities that further limit access to healthcare [3]. Additionally, 20% of participants reported hospital misconduct, such as denial of care, reflecting discrimination within healthcare settings.

Owing to the complex nature of the spread of disease, a combined strategy is more effective. This includes a multi-component strategy focusing on structural (policies, laws, regulatory environment), behavioral (education, counseling, stigma reduction), and biomedical (HIV testing, risk reduction tools, and pre-exposure prophylaxis (PrEP)) interventions. [4,5] While the effectiveness of PrEP is well established globally, PrEP uptake in MSM has been suboptimal. [6] In 2019, of the 1.1 million Americans estimated to have indications for PrEP, only 224,000 received prescriptions [7]. A study in Vietnam concluded that after two years of PrEP rollout, only 10,400 MSM accessed PrEP from an estimated MSM population of 122,000–512,000 [8]. In Pakistan, PrEP uptake is below the target of 30%, with recent evidence suggesting uptake of only 20% of MSM willing to initiate PrEP. [9] Given that MSM remain at disproportionate risk of HIV acquisition in Pakistan, the poor uptake and acceptability of PrEP translates directly into missed opportunities for HIV prevention in this population. [10]

Many barriers to PrEP use among MSM have been identified. These include lack of knowledge regarding PrEP, concerns about the efficacy and safety of medications, stigma associated with HIV, negative attitudes towards PrEP users from other MSM, difficulties in obtaining PrEP in private settings, and concerns regarding disclosing their sexual preferences. [11] However, these studies were conducted in developed countries with social settings that are much different from those observed in Pakistan.

Therefore, it is crucial to understand the barriers to obtaining PrEP, as perceived by MSMs in Pakistan, a conservative country with rising rates of HIV, so that targeted interventions can be developed to address these obstacles and boost the uptake of PrEP.

## Methodology

### Ethics statement

All participants provided written informed consent prior to participation. Interviews were conducted via Zoom, with cameras off and no names displayed for either the interviewer or the participant, to ensure anonymity. This platform was approved for use in the study protocol. Participants were assigned alphanumeric codes (P1–P6) in place of any identifying information. Audio recordings and anonymised transcripts were stored on password-protected systems. The study was approved by the institutional ethics committee (approval number: 2023-8545-24829).

### Objective

To explore the barriers, willingness, and preferred ways of using PrEP among Men who have Sex with Men (MSM) in Karachi, Pakistan.

### Study design

A qualitative study with a descriptive exploratory design was conducted between September and October 2023. The study population consisted of MSM residing in Karachi, Sindh, who were either initiating or already on PrEP. Participants were identified and recruited from an infectious disease clinic at a tertiary care hospital, which provides a range of therapeutic and preventive services including PrEP. Convenience sampling was used, and eligible MSM were approached by their clinician during routine clinic visits and invited to participate. Recruitment continued until data saturation was reached, at which point six in-depth interviews had been completed. As is standard in qualitative research, the sample size was not predetermined.

### Data collection and analysis

The participants were approached by their infectious disease clinician, who obtained their informed consent while they were at the clinic. In-depth interviews were conducted by another team member to minimize power influence in data collection.

Interviews were conducted bilingually in Urdu and English as per the contextual relevance and participant comfort, with a pre-developed semi-structured interview guide until data saturation level was achieved. No new themes emerged after six interviews. The interviews were transcribed and translated verbatim by researcher who is bilingually fluent in Urdu and English languages. De-identified transcripts are provided as Supporting Information (S1 Text). The coding was performed manually through rigorous reviews of the data transcripts by two coauthors independently, after which codes were compared, and any differences were discussed within the team to reach a consensus to refine the codebook. Rigor was insured through inter-coder agreement and audit trail. Thematic analysis was conducted inductively following Braun and Clarke’s thematic analysis approach [12]. This approach comprises six phases: familiarizing ourselves with the data, generating initial codes, searching for themes, reviewing and defining themes, and producing the report. Phrases were reviewed iteratively to generate and finalize consolidated themes.

## Results

### Participant characteristics

Six MSM participated in the study, with a mean age of 34.8 years (range 29–40). The majority were graduates (83.3%) and employed (66.6%), with equal representation from lower and upper middle socioeconomic groups. Most participants reported no substance use (66.6%). Detailed participant characteristics are presented in Table 1.

**Table 1:** Sociodemographic Characteristics of MSM Using PrEP in Karachi, Pakistan, 2024.

	Participants (n = 6)
Age in years, Mean (range)	34.8 (29–40)
Education	
Intermediate	1 (16.7%)
Graduate	5 (83.3%)
Socioeconomic Status	
Lower middle	3 (50.0%)
Upper middle	3 (50.0%)
Occupation	
Employed	4 (66.7%)
Self-employed	2 (33.3%)
Substance use	
None	4 (66.7%)
Cigarettes	1 (16.7%)
Crystal methamphetamine (ice)	1 (16.7%)

MSM, men who have sex with men; PrEP, pre-exposure prophylaxis. Percentages may not total 100% due to rounding.

The data were organized into three predefined thematic categories: (a) facilitators, including factors influencing willingness to access PrEP, (b) barriers to PrEP access, and (c) recommendations to enhance PrEP accessibility among Men who have Sex with Men (MSM) in Karachi, Pakistan. These themes provide a nuanced understanding of the factors shaping PrEP accessibility and can inform tailored interventions to address identified challenges.

### Facilitators and willingness in accessing PrEP

This theme explored the enabling factors that support individuals in seeking and utilizing PrEP. These facilitators included access to and availability of PrEP, awareness of PrEP's preventive benefits, supportive healthcare environments, social support, and the cost of PrEP.

1. Awareness, Access and Availability of PrEP

The availability of PrEP at designated healthcare facilities, coupled with participants’ awareness of these locations, emerged as the most critical determinant for ensuring timely access to PrEP. Awareness and knowledge of the existence of multiple organizations offering these services were identified as key facilitators. Participants emphasized that this information provided them with the flexibility to choose from multiple options that aligned with their preferences and logistical needs. The guidance and clarity provided by their healthcare providers and other community members played a pivotal role in the decision to adopt PrEP as a preventive measure. As one of the participants articulated,

*“I was having a discussion with someone, and the person suggested that I visit [a specialized clinic], because that person also was on PrEP, and the person knew exactly what the procedures are to get it. So, I spoke to that person in detail, and then I followed those steps, and I ended up getting PrEP.” (P.5)*

2. Cost and Affordability

The availability of PrEP free of cost was identified as a significant facilitator in accessing medication. Many participants highlighted that the removal of financial barriers plays a crucial role in obtaining PrEP. Some participants mentioned that they could afford the cost of consulting at a private sector hospital to access PrEP, which was helpful in accessing it. Participants highlighted that accessing PrEP services was perceived as convenient, contingent upon the proximity of the pharmacy, availability of reliable transportation, and financial capacity to afford a ride to the healthcare facility. This underscores the role of minimizing the financial and logistical barriers that could impede its utilization.

*“I go and for every prescription, I pay five hundred rupees (1.79 USD) and get the drugs. 500 rupees is a fee of some sort that I pay for the drugs in 6 months. That is not a lot, and most people can do that without an issue.” (P2)*

3. Perceived benefits and safety of PrEP

Participants highlighted a profound sense of safety and protection associated with the use of PrEP, attributing this to its perceived efficacy in preventing HIV transmission and increasing their willingness to use it. This confidence in PrEP as a preventive measure significantly alleviated their stress and mitigated their fear of contracting HIV. Participants perceived PrEP as a more effective and reliable option for ensuring their safety against HIV transmission than other preventive methods, such as condoms. While acknowledging the protective benefits of condoms, participants highlighted the superior efficacy and convenience of PrEP as key factors influencing their preference. Participants also expressed satisfaction, particularly with regard to tolerability and absence of adverse side effects. One participant expressed the following:

*“You feel happy, and you feel that you have this. There is one less thing to worry about. It actually feels like a shiny new thing. It is something you can take a little bit of pride in. It definitely brings a little relief.” (p2)*

4. Experiences of Professionalism and Non-Judgmental Care in PrEP Services

The participants highlighted that the professional and non-judgmental attitudes of healthcare providers were instrumental in obtaining PrEP. This supportive and empathetic approach fostered a sense of comfort and trust, enabling individuals to navigate the healthcare process with greater ease. The consistent absence of stigma and bias within clinical interactions was particularly appreciated as it alleviated concerns about discrimination, thereby encouraging access and adherence to PrEP recommendations.

*“This could have been very different if my specialist was a different person or had a different attitude. I think I am lucky to have a really good infectious disease specialist.  ... I think that is a remarkable thing to be able to hack that.  ... Then some nurses take your vitals, etc. … I have never felt like I was given a second glance. Then you are at the pharmacy, and nobody has ever looked at me twice or looked …. It has been super professional throughout.” (p2)*

5. Social Support

Social support and partner involvement were identified as key facilitators, as they helped normalize PrEP use within relationships, reduced stigma, and promoted shared decision-making regarding preventive health practices. Additionally, the broader community's acceptance and encouragement contributed to creating a supportive environment that empowered individuals to prioritize their health and overcome barriers to sustained PrEP use.

*“My partner encourages the fact that I am being careful and that I am taking preventive measures. (p5)*

### Barriers in accessing PrEP

1. Lack of Information & Awareness

The lack of information has emerged as a key barrier to PrEP access. Participants considered it essential to have access to trustworthy medical information regarding HIV and PrEP. Since they were aware of HIV risk factors and the availability of PrEP to reduce their risk, they were able to take it consistently. In their view, correct information about HIV/Acquired Immunodeficiency Syndrome (AIDS) and PrEP must be available through widely accessible sources, such as hospital websites and online platforms, so that other community members can also become aware of it. Participants shared their concern regarding general misinformation and misunderstanding that impacts access to information, for example, a general perception that there is no HIV in Pakistan. They also believed that individuals from the MSM community should actively provide awareness and information so that their queries can be answered in a timely manner, which can decrease stress and save time. People in the interviews shared that they considered other individuals with valid information as a resource, which is mostly lacking in our context. As shared by one participant:

“*There are so many other people who do not have such resources available … so it might be an issue for them. But for me, I do have someone who I can go to for prompt responses regarding, for example if there's some information that I need regarding PrEP*.” (P 5)

2. Privacy and confidentiality

Privacy and confidentiality were raised as significant concerns shared by all the participants. Participants were concerned about their identity breach while filling out registration forms or providing any information due to the stigma and discrimination related to HIV and sexuality. They worried about sharing their personal information to access PrEP in the computer system at a pharmacy or appointment counter. Participants living with their family were also concerned about hiding their medicine from family members. Lack of privacy and confidentiality was a major concern at community-based organizations, as participants thought that there was less privacy and confidentiality in comparison to healthcare organizations or hospitals in terms of systems that can control the release of information. One participant shared this by stating:

“*Because going to non-governmental organizations (NGOs) can stigmatize you a bit. Because the people who are operating these NGOs are basically from this community…. because people might know you and they might share this with other people.... Because the community is a very small place. So, you get very stigmatized there*.” (P 6)

3. Financial Barriers

One of the significant financial obstacles reported was the expense of doctor consultations at private-sector hospitals. Participants reported that although medicine is free, paying for required doctor consultations for accessing PrEP in the private sector is unaffordable for many, considering it as an additional and growing expense. Expenses related to diagnostic tests and travel costs to reach the facility further add to the total cost. The cost of obtaining PrEP for a few of them was higher depending on their current financial status. The need for further ongoing medical care exacerbates individual financial problems. One participant quoted doctor consultation charges.

“*That the charges (of doctor consultation) started from Rs.1700 and went up to Rs. 1900. Now, it's nearer to Rs. 4000 (14.31 USD). I don't know about the current. Maybe it's more. The cost really hits the people. The cost in the whole process*.” (Person 6)

4. Logistical & Access Barriers

Participants reported that access to PrEP was impeded by several logistical obstacles, especially those related to pharmacy services. The restricted availability of drugs in pharmacies posed a problem. When they are ready to begin PrEP, access was delayed, because pharmacies do not carry PrEP. Distance and travel to pharmacies and healthcare consultations were the other concerns. For some participants, the limited opening hours of the pharmacy also posed challenges. Participants were unable to visit pharmacies during regular business hours because of employment or other obligations. The participants elaborated on their personal experiences as follows.

“*You get the drugs but sometimes they are short. Last time I went to one of them, and they did not have enough, they were only able to give me one bottle, they took my number, saying they will call me back when they have more. They never got in touch with me. I went back myself; they still did not have it, then I went to the main XYZ pharmacy, they said we only have these two bottles so please take them …. This time it was better, I went, and they were able to give me three bottles, so I was happy. But it’s not always like this. They do fall short sometimes.* “(P2).

*“The issue is that the pharmacy closes at 8 pm, and I need to make sure that I am there to pick it up, I have to go till 7 pm. If I miss that day, then I have to again and plan it for another day. Sometimes, the waiting time is a lot, so that also becomes an issue.” (P5)*

5. Perceived Stigma and Emotional Barriers to PrEP Access

One of the common concerns was the fear of judgment in the healthcare setting. Stigma and perceptions associated with PrEP use and uptake were seen as a barrier. Participants reported perceived fear of judgment in a public healthcare setting. This fear of stigma and judgement leads to emotional health-related concerns including anxiety, overthinking, and anticipated judgment. Since mental health is a concern in PrEP access, emotional experiences can become a barrier to accessing or missing out on receiving a PrEP dose. One participant stated the following:

*“The idea of just getting PrEP to people is horrifying. It's so daunting, but even if you have the money, the means, and just the idea of doing something like that will make it obvious. Because …, going out and getting it is basically you are putting yourself in the system. And it's so daunting. It's so, so daunting to everyone. I remember my first appointment and I was so scared, because you see, it has to do with the country that we live in. It has to do a lot with the way we've been brought up. It has to do a lot with our thinking and the way we think about this kind of life. So, you're going out in public for something which has been shunned, which is something dirty, which is something hidden.” (P4)*

### Recommendations to enhance PrEP Use

This theme encompasses the actionable strategies suggested by the participants to enhance PrEP uptake. Suggestions included increasing community awareness; need for privacy, anonymity, and comfort; preference for alternative PrEP delivery methods; suggestions for improved and streamlined processes; and enhancing support systems for PrEP adherence.

1. Communication and Awareness

The participants recognized significant gaps in awareness and information, especially within vulnerable populations, and highlighted the critical need for targeted outreach and education. The participants recommended leveraging diverse platforms to disseminate information, including dedicated websites, MSM-focused dating applications, and community-based social events. These avenues were found to be effective in discreetly and efficiently reaching key populations. Furthermore, they underscored the importance of integrating comprehensive sex education into awareness campaigns, emphasizing that education should not only convey the benefits and use of PrEP, but also challenge the stigma surrounding HIV prevention methods. As one participant articulated,

*“I feel like the information needs to be where people are, for example, dating/hook-up applications.  ... Information issues and misconceptions are there.” (p2)*

2. Privacy, Anonymity and comfort

Privacy, confidentiality, and fear of stigma and discrimination were identified by the participants as critical barriers to accessing HIV prevention services. To address these challenges, participants recommended introducing online consultation platforms and separate clinical setups that would allow individuals to discuss personal and sexual health concerns in private and anonymous settings. Given the sociocultural context of Pakistan, where discussions about sexual health remain taboo, MSM participants expressed a preference for male health care providers. They emphasized that having access to providers of the same gender would facilitate open communication about sensitive sexual health issues. Such culturally sensitive and privacy-oriented interventions were viewed as essential for overcoming existing barriers and improving the uptake of HIV prevention services.

“*There are people who may not be comfortable going to a clinic and sitting face to face with a doctor and talking about their personal life. So, I think online will be a good … so they can anonymously share.* ” (p1)

3. Preference for Alternative PrEP Delivery Methods

Although most participants expressed satisfaction with their current PrEP regimens, some highlighted a preference for long-acting injectable options or methods that could offer extended periods of prevention and safety. This preference stems from the desire for increased convenience and reduced reliance on daily adherence. Participants who used PrEP on-demand emphasized the advantages of medications with a longer shelf life, noting that such options would reduce the burden of frequent renewals and mitigate concerns over medication expiration.

“*If injectables are available and you have to get injected once a month, that would be good too because doing it once a month would do the job.*“ (p3).

4. Improved and streamlined process

Participants highlighted significant challenges associated with renewing PrEP prescriptions every six months. This process, which requires multiple steps—consulting a doctor, undergoing HIV and related health tests, and obtaining medication—was described as time consuming and inconvenient. Long waiting times for appointments, tests, and results add to this burden, making it difficult for individuals to fit these activities into their busy routines. The participants suggested streamlining the process to save time and effort. Furthermore, participants suggested expanding services to include home delivery of HIV tests and PrEP medications. This approach was viewed as a practical way to maintain discretion and improve accessibility. One participant emphasized this by stating,

*“It's such a long process. Consulting the doctor, testing, and then getting it from the pharmacy. Then those who have to take it skip it. Or you can offer a package where all three or four things can be offered at the same time. Or a specific day when you can only address these people. (P.6)*

*“So, if there was a way that they could get PrEP at home just like XYZ lab comes to your home to do tests, similarly this system could be there.”* (p3)

5. Enhancing Support Systems for PrEP Adherence

Participants frequently expressed concerns about understanding how to properly take or adjust their PrEP regimen. These uncertainties often arise during moments of confusion or worry, highlighting the need for timely and accessible support. Many participants emphasized the importance of having reliable resources to provide guidance and reassurance, particularly when they were unsure about their medication schedules, side effects, or the process of pausing and resuming PrEP. Beyond practical information, some individuals sought emotional validation for their concerns, while others needed assistance in navigating healthcare systems to access or retain PrEP services.

*“There should be a PrEP helpline and one for daily concern sharing too. Anything unusual or doubtful comes to your mind, you can share with the person who can validate your concerns as being right or wrong. (p3)*

To address these challenges, participants recommended strategies such as establishing helplines, digital tools, or access to well-informed healthcare providers who could offer clear, immediate answers, and empathetic support. These measures could help ensure consistent adherence and alleviate stress associated with PrEP management.

## Discussion

The availability of PrEP has created a substantial opportunity to reduce HIV risk in vulnerable populations. However, its uptake is still not optimal for the MSM population of Pakistan. This study suggests that MSM are willing to use it, and its availability is considered a useful tool by those who use it. The study reported feelings of safety and relief from stress (of getting HIV infection) with the use of PrEP. The lack of side effects not only contributed to users’ comfort, but also reinforced their confidence in PrEP as a viable option for HIV prevention. However, the findings also highlighted significant challenges in accessing PrEP, including a lack of information and awareness among community members, concerns about privacy and confidentiality, financial challenges, and logistical issues. These findings are consistent with past studies on PrEP accessibility, highlighting the structural and systemic barriers to its uptake.

Previous studies have reported similar results. A systematic review conducted in low- and middle-income countries (LMICs) reported high levels of willingness to use PrEP yet low levels of awareness among MSM [13]. Another systematic review conducted on global data also suggested the importance of awareness of PrEP among MSM to increase its uptake [14]. Information about sites provides clarity and assurance about where to obtain PrEP, minimizing logistical issues. This finding is supported by research from South Africa, which showed that establishing specialized PrEP clinics inside sexual health services improves accessibility and provides continuity of care [15]. Increasing the number of such facilities, especially in underserved areas, can help to increase reach and equity. Previous studies recommended using digital media, tele-health helplines, relevant social gatherings, community health programs, and peer education to bridge this information gap [16–18].

This study further identified privacy and confidentiality as major concerns for MSM due to the stigma related to HIV and sexuality. Similar findings have been documented, where stigma and discrimination discourage people from using HIV prevention services and are less likely to use PrEP [19]. Overcoming this barrier requires ensuring privacy and enabling secure, anonymous, or discrete access to PrEP services through online consultations and home delivery. Community-level initiatives that normalize HIV prevention discussions can reduce stigma and increase trust in health care systems [20]. Perceived stigma and fear of being discriminated against significantly affects the initiation of PrEP, which becomes a barrier [21]. The professional and non-judgmental attitudes of healthcare providers were identified as critical. Judgmental attitudes or discriminatory behaviors among healthcare practitioners discourage people from seeking care, especially for sensitive issues, such as HIV prevention [22,23]. This study also reported a male gender preference in consultations due to the cultural sensitivity of the local context.

Free of cost and the regular availability of PrEP at specified and proximal healthcare facilities have emerged as critical facilitators of PrEP utilization. Studies from other settings have also suggested the importance of providing PrEP free of cost to increase its use. According to a study conducted in Kenya, financial constraints were a major factor in PrEP discontinuation among at-risk populations [24]. Another study from India also reported cost and affordability to be major concerns [25]. Financial burden has been categorized as a structural and system-based barrier for MSM populations in various studies, including high-income settings [10,26]. A consultant appointment in Karachi, which is a requirement to obtain PrEP, can cost up to 16–18 USD in the private sector setting, and the required blood tests and travel to the health care center adds further to the cost. However, those who could afford it preferred the private sector because of their professional and confidential services.

The study also identified inconvenience due to other systematic barriers, such as long waiting time for consultation, waiting for the results of the required test reports, and waiting time at the pharmacy. This often required multiple visits during working hours, which was difficult to manage. PrEP uptake in the vulnerable population of Pakistan will require reducing systematic barriers and ensuring privacy and confidentiality to reduce the fear of stigma and discrimination. Addressing these barriers will require service models shaped around the preferences participants voiced, including discreet online and home-based delivery, male service providers, and decentralized access through multiple pharmacies.

This study has several strengths. To the best of our knowledge, this is the first qualitative study in Pakistan to explore barriers, facilitators, and preferences around PrEP use among MSM, addressing a significant gap in the literature. The qualitative design provided in-depth insights into a sensitive and under-researched topic that quantitative methods would not fully capture. The findings offer context-specific perspectives from a high-stigma, conservative setting, contributing to a literature that is largely dominated by high-income country data. The use of anonymous Zoom-based interviews with cameras off provided a safe and private space for participants to discuss sensitive issues, which may have facilitated more candid disclosure.

However, we also acknowledge several limitations. The sample size was small and limited to MSM already engaged in PrEP services at a single tertiary care facility in Karachi, which may not reflect the experiences of harder-to-reach MSM or those in other parts of Pakistan. Recruitment through an infectious disease clinic may have introduced selection bias, as participants were already engaged with healthcare services. Clinician-linked recruitment may also have introduced social desirability bias, with participants potentially more inclined to report positive experiences of care. However, the use of anonymous Zoom-based interviews with cameras off may have partially mitigated this.

Future research exploring the perspectives of MSM who are not yet using PrEP would be crucial in identifying barriers from a different vantage point. It would also be worth exploring the level of awareness among the broader MSM community in Pakistan.

## Conclusion

To the best of our knowledge, this is the first qualitative study in Pakistan to explore the barriers, facilitators, and preferred ways of using PrEP among MSM. While PrEP is available through existing programs in Pakistan, no formal policy currently guides its delivery for MSM specifically. This study emphasizes the importance of structural, social, and interpersonal factors in increasing PrEP uptake. Raising awareness through digital platforms and MSM social networks, ensuring privacy and confidentiality, and streamlining the access process at minimal time and cost are key recommendations. Services should be adapted to the needs and comfort of MSM, including male service providers, online consultation and helpline services, home testing, availability of PrEP across multiple pharmacies, and home delivery of PrEP.

## Supporting information

S1 Text De-identified in-depth interview transcripts. Verbatim transcripts of the six in-depth interviews with men who have sex with men (coded P1–P6) that constitute the qualitative dataset underlying this study. All direct identifiers (participant and provider names, institution names, and contact details) and indirect identifiers (specific dates, city-level locations, body metrics, occupation, and other potentially identifying descriptors) were removed or generalized in accordance with participant consent and the journal's data-availability policy.
(DOCX)
