# Supplementary material for: “One less thing to worry about”: Barriers and facilitators of PrEP uptake among MSM in Karachi, Pakistan: A qualitative study
Source: PLOS Glob Public Health. 2026 Aug 28;6(8):e0007151. doi: 10.1371/journal.pgph.0007151 (PMC13524246; doi:10.1371/journal.pgph.0007151)
Supplement: Supplementary file 1 [file pgph.0007151.s001.docx]

PrEP Interviews - Combined Document

## Interview 1

Interviewer:

We are doing this study to see what barriers and challenges people are facing when accessing PrEP particularly and your participation in this study is completely voluntary. The questions and answers will be recorded in the meeting.

No one except the interviewer and principal investigators will have access to the recordings and once transcribed, no identifiers will be used. Your participation anonymity will be kept, and it will be confidential. If at any point you feel uncomfortable or you want to withdraw from the study, your choice will be respected.

We will send you a digital copy of the consent form which you will have to sign and send back to us. If it is clear, we can get started?

Participant:

I think everything is clear.

Interviewer:

Okay perfect. Just as a startup question. What information and knowledge do you have about HIV prevention?

Participant:

So I know that using condoms is a safer way to have sex and for prevention I know PrEP. So safer sex and PrEP. And also not having multiple partners because sometimes I hear that which is more of a behavioral thing.

Interviewer:

Which step do you personally take for HIV prevention?

Participant:

Safer sex and PrEP.

Interviewer:

With regards to PrEP, what do you generally know about PrEP?

Participant:

So generally, I know this main fact that it helps in preventing HIV even if you do not have safer sex and if your lifestyle is a little promiscuous when you have multiple partners even though you are practicing safer sex by using condoms from time to time. Sometimes at the heat of the moment you forget about it or avoid it and don’t use it, so it is always better to have a secondary precaution which is taking the medicines to be more on safer side. PrEP I do not know how to explain, but the general knowledge I have is that it helps in preventing HIV.

Interviewer:

How long have you used PrEP for?

Participant:

So I started using this in year in [a month] or last year in [a month], I am forgetting exactly when. No no, it was actually before that. It was [a previous year], sorry.

Interviewer:

Okay, [a previous year]. So close to a year now?

Participant:

Yeah.

Interviewer:

Okay. And where do you get it from? Where do you access it?

Participant:

From [tertiary care hospital]

Interviewer:

What has your experience so far been with using PrEP?

Participant:

I do not use it on a daily basis. I use it as a periodical thing when I am more active. Usage wise it ha been okay. I was informed about the side effects which were nausea and if you use it regularly then you might need to get your kidneys checked for stones. So, I preferred using it periodically and it is working fine for me. I did not feel any severe side effects.

Interviewer:

In the past year, have you ever felt or though to stop taking PrEP?

Participant:

No, I haven’t.

Interviewer:

Okay, and you access it from [tertiary care hospital]? Correct? And how do you commute to [tertiary care hospital] to get PrEP or has that ever been an issue for you?

Participant:

Yeah. No, never an issue has I have my personal conveyance. Whenever I am in [a large city in the country], I drive by myself. It is easier for me to go and get it.

Interviewer:

Has cost ever been an issue for you when accessing PrEP?

Participant:

No, its quite nominal Rs 500 fee for 3 months of medicines. So I think it is perfectly fine.

Interviewer:

Generally, are you comfortable and satisfied with the process that you have to go through to access PrEP?

Participant:

Yes. Initially I had a bit of a difficulty to identify who to meet in [tertiary care hospital] in order to get PrEP. All I knew form one of the friends or acquaintances that [tertiary care hospital] is now giving PrEP as well. So when first time I tried to access PrEP, I actually went to a General Physician. I spoke to her very openly and in a confidential manner and she said okay, I am hearing it for the first time as well. She is a consultant in [tertiary care hospital] but she was a General practitioner so she said “I am not aware of PrEP. Let me find out what it is and what is it used for.” Right there and then when I had my appointment ongoing with her, she went on a call with some other doctor and then she found “Okay, you need to meet Dr ABC for it and for that you have to book another appointment.” So that is how I met Dr ABC and booked the appointment and got access. So initially it was a bit difficult in order to figure out who exactly to go to because the website also doesn’t really give much information when it comes to accessing a specific kind of medicine or treatment. It was a tried and tested kind of process.

Interviewer:

And apart from [tertiary care hospital], do you know about the availability of PrEP otherwise? Generally in the country or other places?

Participant:

I know the testing centers they have for example; ‘[community NGO]’ for male health alliances. These test centers have access to PrEP as well but over there I know that first you have to go to the test center, then they link you to a hospital so the process over there seems a little bit tedious to me. But I know these [community NGO]-run HIV prevention centers have access to PrEP as well.

Interviewer:

In your opinion, what do you think is the ideal way to access PrEP?

Participant:

In my opinion? You mean consulting with the doctor and all?

Interviewer:

Yes, so the process you are going through right now, do you feel comfortable? Is there any other way you think would be better or is there anything else which you think would be an ideal way to get PrEP? Could be things like home delivery or online consultations. Anything which you think will make this process a lot easier or would be a better alternative than what you have to go through right now.

Participant:

So personally speaking, I think I am comfortable with the process that is there right now other than the fact that I think there should be more information with the people who are booking appointments for the doctor or on the [tertiary care hospital] website. Like I said earlier, I had to go through a General Physician in order to figure out which doctor I need to talk to about PrEP. So, if that part can be communicated more clearly to the people who are trying to access it, it will be easier. But then after that, the process is very simple and easy. Additionally, knowing from my friend circle that some of my friends are not comfortable going to the doctor and talking about their lifestyle and everything. I spoke to one of my friends on one on one basis and he said “I went for HIV testing to one of these centers and I never told them I was gay and I wanted PrEP because I have sex as well as I am a little bit shy and I am not comfortable sharing this information so if you have the medicines, why don’t you share it with me?” I said I can understand your shyness. But my point is that there are people who may not be comfortable going to a clinic and sitting face to face with a doctor and talking about their personal life. So, I think online will be a good initiative for these kinds of people so they can anonymously share. But I don’t know how that works. I think online will be helpful for people who are not comfortable going to a doctor physically.

Interviewer:

Thank you for sharing that. From what I can gather getting information was difficult bit for you personally. Right?

Participant:

Yeah.

Interviewer:

Do you think there needs to be a specific helpline or a source of information that you might have wanted to connect to before having to go through all this process? Regarding services and general information?

Participant:

Yeah, I think that will be really helpful. Someone like me who is very out and about had to figure out who to speak to in order to find out a way for consultation and PrEP. An online platform or a hotline which can be accessed easily will definitely be helpful.

Interviewer:

And with regards to who would you like to connect to, is there a specific type of person or level of profession? Like would it only be doctors you want to connect to or would you like to be connected to anyone in the healthcare profession or anyone in the support system profession?

Would there be a specific person or job description that you would like to connect to or was it okay that there was anyone on the other side that is compatible enough to give you this information?

Participant:

Personally, I think I will be more comfortable if it is a doctor itself and not an assistant or anybody else. Because I remember that the first time when I came to see Dr ABC, I was more comfortable because I was talking to the doctor although his assistant was also there at the same time. But it crossed my mind at that time that if I was talking to the young assistant, I think I would not have been much open or comfortable sharing information compared to being in front of a professional doctor.

Interviewer:

Of course, understandable. So currently, what are your sources of information which you are turning to?

Participant:

Dr ABC right now, the one I consult with.

Interviewer:

Currently, are you taking the tablet form?

Participant:

Yes

Interviewer:

Is there any other form that you would like PrEP to be available in?

Participant:

No, I think tablet form is fine. Do you mean like a syrup? I don’t think that will work. I think tablet is easier.

Interviewer:

Long term injections or implantable devices. Anything else that you think would be easier to access apart from the tablet form that you are already taking?

Participant:

I think it would be easier if they found a stronger version of it like you have to take one pill a month or maybe you get an injection once.

Interviewer:

Something longer acting.

Participant:

Yeah, something which has a longer time period to cover instead of taking it on daily basis. That will be helpful.

Interviewer:

Currently, do you receive any social support regarding accessing PrEP or being a part of this community. Family? Friends? Organizations?

Participant:

Social support is there. Some of my friends also started taking PrEP at the same time that I did, and we didn’t share information with each other. Each of us individually went out and tried to find out how to get it. Once I had a party and we were all sitting down and someone said, “Oh you know I am also taking PrEP.” So, there is a support system and there was a discussion for people who are not aware of it to let them know about it and make them comfortable and motivated to go and get it. It is always better to be on safer end. So yeah, support is there in the community but not from family side because as much as I am open, I do not share such information with my family on that level.

Interviewer:

Do you think generally there is a lack of support not particularly, but do you think that other people in the community might seek out support system and might not be able to get it?

Participant:

No, I think that is lacking in [a large city in the country]. I am speaking for [a large city in the country] specifically; I don’t know about other cities. You mean support as in like professional support or friends and family?

Interviewer:

Social support or moral support from family, organizations or community?

Participant:

No, I think we are still lacking that a lot. Something needs to be done in terms of communication and information so that people at least know who to go to and get further information if they are interested.

Interviewer:

I am going to ask you specific questions regarding potential barriers you encountered or might not have encountered. These are open-ended so you feel free to speak from your own experience. When you are accessing PrEP currently, do you have any concerns regarding your confidentiality and safety?

Participant:

No. that is the reason I prefer [tertiary care hospital] over these other small [community NGO]s because I feel that over there the data leak can be on a much higher risk compared to a professional place like [tertiary care hospital]. So no, I feel comfortable going to [tertiary care hospital].

Interviewer:

Right. What have your experiences been with people you have interacted with while accessing PrEP? So nurses, physicians, residents or medical students that may have been around?

Participant:

So far it has been good. Nothing to point out or feel that this is not comfortable.

Interviewer:

Alright. And what has your experience generally been with the pharmacists when buying PrEP?

Participant:

Personally, I always wondered if they know what it is being used for and judge me for it. Otherwise generally, I have been comfortable accessing it even at the pharmacy after the prescription is handed out.

Interviewer:

Because you mentioned judgement, have you ever felt stigmatized or judged while accessing PrEP?

Participant:

Not really. I do wonder if they know and what they might be thinking but I have not been at the receiving end of any such behavior.

Interviewer:

Okay, what do you think are the perceptions or general feelings about being connected with somebody. If you are on PrEP, has there been a difference in connection with your partners or has that been an issue if the other person is not on PrEP but you are on PrEP?

Participant:

I am sorry I did not understand the question.

Interviewer:

Has being on PrEP changed your feelings/perceptions about you when you are being connected with your partner? Has that been an area of problem, discussion or concern generally?

Participant:

No, I think PrEP has actually helped me feel more relaxed and not be afraid in my head all the time when I am having intercourse.

Interviewer:

Have there been time related limitations of receiving PrEP? Delay in delivery of medication or the fact that you have to take the medication a couple hours before engaging in intercourse? Has that ever been a difficult routine to follow?

Participant:

So accessing the medicine the medication hasn’t been an issue but yeah, the timeline thing is something I kind of feel difficult to follow very strictly. So sometimes I forget to take it at the right time and then obviously I message my doctor asking. The recent conversation was that I forgot to take it at the right time and then I remembered after 7 hours that I was supposed to take the medicine, so I immediately called my doctor and asked him if it is okay or do I have to take another two tablets again or something. The time thing is a little difficult for me to follow through, I don’t know about others.

Interviewer:

Is there anything you would want to recommend or suggest regarding prevention or PrEP availability which according to you would make a difference to the community or your health generally? Any suggestions about it?

Participant:

My suggestion would be to have the communication more out there and more clear because there are a lot of people who are either ‘bi’ or ‘gay’ I feel but being in the community are too shy to access it or sometimes they don’t even know. I feel it should not be a taboo to talk about it, it should be discussed or at least communicated openly om official channels so that people can be more aware of it and have access to it.

Interviewer:

In your opinion, who do you think is responsible for provision of PrEP or providing information about PrEP?

Participant:

I think the hospital management should look at it. I am not going to say government because you know there are elements over there which make you avoid such things to be discussed publicly. So, I think if hospital managements take more proactive measure where they treat this lifestyle as not a sinful lifestyle and something we should not be talking about because we live in Pakistan. If they treat this thing from a medical perspective and make sure that the message goes out, I think that would be more helpful.

Interviewer:

Thank you so much for sharing that. I think that is it for questions from my end. Do you have any questions?

Participant:

So, this research is for what purpose? If you don’t mind elaborating.

Interviewer:

Of course. So we are trying to understand what barriers the community is thinking when accessing PrEP and we are hoping that once we have highlighted that some people might not even know that PrEP exists and once they find out they have no ways of figuring out where to get it from. And for some people time and conveyance or financial reasons (in people who do not have a stable source of earning themselves) might come into play when accessing PrEP. So we are trying to highlight exactly what issues the community is facing when accessing PrEP and once we have that, we are hoping to propose suggestions/solutions to the issues. The end goal is to make sure that PrEP availability is enhanced, and it is accessible to the population as needed because medicine itself has proved to be very successful in preventing HIV. We are hoping somehow we can improve its access.

Participant:

Okay. I have two more suggestions while I was listening. They just popped up.

Interviewer:

Yes, of course.

Participant:

I think it would be very good if you have some advocates from the community. I am speaking specifically about [tertiary care hospital]’s objectives if you really want things to become smoother for the community to access PrEP. I think having advocates, somebody who is in and out of the community a lot and can access to people who can distribute information. I know personally that there are gatherings and parties, and it will be a good starting point to spread the word out. Secondly, probably getting the messages across on these dating apps which they use usually. Especially the pop-up messages which show in Urdu would be helpful as well to spread the word out and make people realize that this is something accessible as well in order to prevent HIV.

Interviewer:

Yeah, I agree actually. Thank you. Any other questions you may have for me?

Participant:

You might know better since it is your field. I just want to know for PrEP, are they working on stronger medicine or other variations of this since you asked me that. I am just curious.

Interviewer:

The ones we have currently come in multiple combinations. We try and see which combinations work best for each patient because not everybody tolerates every drug. I am not really sure if they are working on newer drug.

Participant:

I was just curious since you asked me if there was another form you would prefer instead of tablet form.

Interviewer:

Dr ABC would be the best person to ask this from because his knowledge surpasses mine. Dr ABC will forward you the consent form so if you could digitally sign it or manually sign it and scan it, send it back to us so we can keep it for our record. Your identity will be kept completely anonymous. Of course this participation is completely voluntary and at some point you wish not to be a part of it you can let us know.

Participant:

No, I am glad you are doing this research. I hope something fruitful comes out of this.

Interviewer:

Yes, we hope so too. If you think there is anyone in the community or your circle who would be comfortable talking to us or being part of the study, you can always tell Dr ABC and we will be happy to recruit them as well.

Participant:

Sure, I will let Dr ABC know if any of my friends are interested. I will spread the word that I interviewed for this research and if anybody else is interested I can get them in touch with Dr ABC.

Interviewer:

Yes, that would be really helpful. Thank you so much for your time.

Participant:

Thank you.

## Interview 2

Interviewer:

I will take your verbal consent, once the interview has been conducted, we will talk about the written consent as well. Do you give consent for this interview?

Participant:

Sure.

Interview:

Thank you. My first question would be, what do you know about HIV prevention?

Participant:

Well, for the larger part of our lives, we learnt about safer sex and condoms were the only means that we knew about and as of the past I do not know how many years, as we learned about PrEP, then about 2-3 years ago we learnt that we had access to PrEP. If you ask me a more specific question, I could perhaps give you a better answer.

Interviewer:

So, I think this takes me to another question that is PrEP the only way of prevention for HIV?

Participant:

No, of course not, you can always have protective sex and HIV is also a larger problem in Pakistan. It also affects children. PrEP only deals with people who may get HIV through sexual means from what I understand. If children get HIV out of infected syringes, there is no way they would have been on PrEP. I think PrEP has a limited target audience.

Interviewer:

So, the next question is particularly about your own experience. Which steps do you take for HIV prevention?

Participant:

Well, two things like condom and PrEP. I have taken PrEP at various times in both its forms in terms of being on PrEP on a daily basis versus also being on PrEP on a shorter/on demand regimen. In addition to that or as an alternate, I have always used condoms. Does that answer your question?

Interviewer:

Yeah, I think that answers the question. So, I think when you talk about PrEP, I will now specifically discuss some of the questions regarding PrEP. What are the concerns regarding confidentiality and safety while accessing PrEP?

Participant:

Well, I did this a long time ago but while accessing PrEP, you have to be on a program. I do not know if you have seen this, but I am sure you have, the forms you have to fill in earlier on to be able to get PrEP, for myself I did it and I was happy to do it. But that was a difficult thing because as a person who is not in medicine and who does not work in public health, you don’t know who these organizations are and where this data is going and who might have access to it. I am also very skeptical of things that are free. I really like that PrEP is available for free, but it is an unfamiliar thing for us. You are also giving up a lot of data for it. This is definitely a bit of a concern but then again, I did this 3 years ago, so I do not remember how I felt filling that form, but I do know that for other people the application process and form filling is something that is a concern. I know at least one person from our conversation who did not get PrEP because he did not want to fill that data information.

Can you repeat your question? I feel like I have forgotten your question, and I am going somewhere else.

Interviewer:

My question is what are your concerns regarding confidentiality and safety while accessing PrEP?

Participant:

My situation for the person that I go to for PrEP, I have no confidentiality concerns per say. But that’s me as I generally have fewer confidentiality concerns. That does not indicate the experience of other people. I feel like the situation is not ideal.

Interviewer:

Maybe you want to specify about yourself, any one or two concerns about confidentiality or safety maybe?

Participant:

I will give you specifically my own example. I go to [tertiary care hospital] to access to get my prescription/ to get my consultation regarding PrEP. I also live in that neighborhood. Every time that I am at [tertiary care hospital], or I am at the pharmacy, I run into a neighbor of mine. That’s generally not a concern because people are not rude enough to ask you what you are there for. You get worried a little bit about what if they ask you what you are there for. If they are getting their prescriptions filled at the same time as you are then every time, I am at the pharmacy getting my prescription, there is some commotion and confusion, and people do not know what to do. The instructions are flying from person to person, I really wonder whether these people know what these drugs are. I do not get a sense of judgement from them, speaking definitely. But it is a bit of a concern, I can imagine that something could be improved. The data that was collected earlier, I still wonder where all that data went because it had everything about me on it. I do not know what that was for and where that went or where I might see it again.

When I go to a doctor or infectious disease specialist, that is very generic and could be anything. It is not someone who specifically deals with STIs. That part is okay. There is a little bit of room for improvement in getting the PrEP business and could be a smoother process.

Interviewer:

Thank you for sharing that. This draws me towards discussing what are your experiences with using PrEP? So, you said that you have used PrEP, what are some of the relevant experiences you want to share?

Participant:

For the drug itself, in terms of my physical experience of using the drug is that I deal with it quite nicely like I never reacted badly. It has never caused any negative side effects that I know of. It has never made me feel sick, nothing. In terms of actual use of drug, I cannot tell you of a negative side effect. I think for PrEP in demand, you have to make sure that you have the drug in hand when you need it. If you are somewhere else and your drugs are somewhere else, it does not work. That’s definitely a situation which has happened to me where I needed to take PrEP and I was somewhere else while the drug was at home. Did you mean something else in terms of experience of using the drug or did you mean the actual physical experience? Did I answer your question?

Interviewer:

It was very open-ended so you could share anything. I think we have talked a lot about that you have got PrEP, you have used it. How long do you use it and where do you usually get this from?

Participant:

I cannot tell you how long I have used it. I think I started using it at [age withheld] maybe. I am not sure. I have always only gotten it via prescription from [tertiary care hospital] and I have only picked up the drug from [tertiary care hospital] pharmacy. I have gotten it both from [tertiary care hospital] and [tertiary care hospital]. I have not gotten PrEP from anywhere else, or a prescription or a=information/anything from anywhere else.

Interviewer:

So, if you have gotten it from [tertiary care hospital], have you ever felt that commuting and costing would be an issue? Any problem in paying the cost or buying?

Participant:

The problem is not getting the PrEP itself, the transport I can imagine could be a problem for other people. For me, I literally live walking distance from [tertiary care hospital] so that is not a problem for me. The drug itself, you are paying a nominal Rs 500 fee which is a service fee. Other than that, the drug itself is free. However, when you are on these programs, you are also seeing a specialist and that’s Rs 4500 and then you would be written tests which you will get there and then and that is going to be another Rs 4500. So, if you mix all of these up, every one of these trips that I take twice a year, it costs Rs 10,000. So, it’s not the drug itself, but the peripheral stuff becomes expensive.

Interviewer:

Okay. As you have mentioned that you have only been getting the PrEP from [tertiary care hospital] or [tertiary care hospital], what do you know about availability of PrEP? Do you think that it is available somewhere else too?

Participant:

I was trying to find something which would answer one of your earlier questions. I am aware of PrEP being available elsewhere. In fact, before I learned that PrEP is available at [tertiary care hospital], I learnt of other organizations and NGOs that were providing PrEP. I cannot recall their names right now. I definitely know about PrEP being available elsewhere and to other people from other organizations that deal with more focused communities.

Interviewer:

Are you personally satisfied with the way you are receiving PrEP right now?

Participant:

I am going to answer your earlier question. You asked me when I had started using PrEP, I had tried to get a prescription back in [year] and that time, you needed to get two drugs for PrEP to use together which I could not find. One was available, one was not. This was my first attempt at getting PrEP. The prescription also came from an Infectious Diseases specialist at [tertiary care hospital]. This could not happen. The first time I actually got my prescription as well as drugs was [month/year].

What was your question now? If you could repeat.

Interviewer:

I will come back to the other question but the discussion you have started brings me to one more question. Why do you feel you were unable to access those specific medications?

Participant:

Because I could not find them anywhere.

Interviewer:

Was there an issue of availability?

Participant:

I should clarify that at that time, PrEP was not available as a single pill. It was available as a combination, so you had to get two drugs and have them together as one thing. I still have that prescription, one was available everywhere and the other, which was also used to treat hepatitis I guess, I looked for it everywhere be it [tertiary care hospital] or [tertiary care hospital] or [a local pharmacy]. Nobody had it. At that time, we did not have the combination pills which are available now. We had to buy the combination drugs and we could not get one of the two components as it was unavailable. This was back in [year]. From [year], combination pills were available I went to the same person, and I got the prescription, so I was able to buy it from [tertiary care hospital] pharmacy.

Interviewer:

Was somebody instrumental and someone you would have liked to connect with for information and services regarding PrEP during this experience? In order to get help and know regarding services?

Participant:

I have a comfortable relationship with Dr ABC. But I can only talk to him when I visit him generally. I really did not have the need for sudden information for something, so I do not know how to answer that. I have never had a situation where I felt like I really needed to find out about this and who to contact for it. I will say that you do need access to someone you can call and ask things perhaps anonymously that you need such and such information, to explain something or to answer a question. If something is semi critical or you are curious about it or concerned that you might do something wrong then you do not want to wait for however long, travel and meet them for a consultation to ask them a simple information-related question. It would be nice if something were available like calling someone and asking. I do not have that right now. I could perhaps message Dr ABC but that is not appropriate and that is not his job.

Interviewer:

Thank you so much for mentioning this. I want to go back to the information you said about having two drugs back in [year]. I really want to inquire if you felt that there should be a specific form of PrEP available. Should it be a tablet, an injection or any other form? Have you ever thought about it?

Participant:

The PrEP is already a pill, I cannot imagine anything simpler. If someone told me to get an injection that would last a month, that would be easier for me. But I believe pills are easier for people compared to other forms. I am quite happy with this situation because I don’t have to follow the everyday situation because that feels a little excessive that you are taking a drug every single day on the off chance that very rarely you would be in a situation and be like it would have been nice if I was on PrEP. Being able to take it on a shorter demand regime is really ideal for me and I cannot imagine a simpler means of having it. These drugs were magically given to me more easily. I do not want the drugs delivered to me instead I actually want to travel and get them myself.

Interviewer:

This takes me to the discussion we started in the earlier phase. Tell me, are you satisfied with the overall way you are accessing PrEP? Based on the experiences you have shared with us.

Participant:

Yeah, I think so, for myself I am pretty all right. It’s very difficult to think that you are going to [tertiary care hospital] and getting this wonderful smooth service etc. So, I feel satisfied, but I think most people do not have access to it. I can say this for myself, but our experiences are colored that you have access, can travel to these places and pay consultation fee, pay for tests and get the drug. I am aware that I can do this but it’s not necessarily available in the same way to other people. It’s a concern that you also have to see a consultant. I am thinking of similar situations where you are sitting and waiting for your appointment, and you run into relatives or friends. They ask what you are here for, and you say I am here to see an infectious disease specialist as you have nothing else to say. They ask what happened to you. So, it becomes complicated in that sense. These are things that do not necessarily affect me so much because I am older and independent. I know how to lie to people and navigate situations, but these are definitely concerns to a smaller degree for myself but to a much greater degree for other people. In other ways, I am happy, and it is easy enough. You get the drugs but sometimes they are short. Last time I went to one of them and they did not have enough, they were only able to give me one bottle, they took my number saying they will call me back when they have more. They never got in touch with me. I went back myself; they still did not have it then I went to main [tertiary care hospital] pharmacy, they said we only have these two bottles so please take them because these won’t be available after this time. I have a prescription in my hand which mentions three bottles, so I think I should not be doing so much processing to get a prescription pill especially when they have it. This time it was better, I went, and they were able to give me three bottles, so I was happy. But it’s not always like this. They do fall short sometimes. We live in Pakistan after all, and we have kind of sense of humor for these kinds of situations. Thankfully, it’s great for me.

Interviewer:

I think there is no right or wrong answer as we are talking about your experiences, so it is open-ended. We have been picking up multiple things from your answers, but I just want to clarify one thing, do you have any ideal ways in mind of getting PrEP? Do you think there is an ideal way in which PrEP should be available?

Participant:

Do you mean it in terms of the form of the drug or in terms of how I get access to it? Do you mean if it is in pill form, how do I get better access to it? Is that what you mean?

Interviewer:

I think you have already discussed the form of PrEP. Here we are actually talking about the ideal way of access.

Participant:

Getting the drug itself is what I am alright with. Having to see a consultant to be able to get the drug, I think that becomes a little bit of an impediment. This makes the process very large. I have done this twice or thrice, there is newness so I am happy to do it, but it will become redundant at some point. I do hope that at some time, there is a point of contact who tells me that you are due for an HIV test or kidney function test, it has been six months, get the tests done and send them to me and go and pick the drugs from the pharmacy. That would make things quite easy for me. This includes all those things which you can do at your own time and convenience. I understand that there are some conditions like they do want you to make sure that you are tested, they want to make sure that once a year your kidneys are working all right, that you don’t actually have HIV. If the process of getting a prescription could be better and simpler, the process of getting the drug would be pretty smooth itself.

Interviewer:

So, I think speaking of time that you said, and you shared the ideal way, were there any time related limitations or do you want to talk about any specific limitations of receiving PrEP which are associated with time?

Participant:

One issue is that the PrEP we are given, so I have a bottle I front me right now which was manufactured in [year] and it expires in [month/year]. Because I take PrEP on demand, I am going to put a bunch of these in trash eventually because these might not be used and expire. If it expired, I would obviously throw it away and not use the expired drug. If there was a longer expiration date on these drugs, it would have made my life much simpler. Because the pharmacies do not know how to process it, they cannot give you an on-demand prescription but for a set duration. I took the 3-month prescription to them, and they gave me three bottles. But these bottles are now expiring after 3 months. I would end up wasting a lot of these, if these had a longer expiration of for example after a year so these three bottles could have lasted me a year. This would make my life easier, and the drugs would not be wasted. This is not just this time; this has happened every single time. The expiration on the drugs is very short and this is my big peeve with these bottles of drugs. I cannot extend their use.

If you need to see a consultant, then you have to wait till you get an appointment. When you get there, there is a lot of peripheral activity that happens. Your vitals are taken. Last time I went to see my infectious diseases specialist, I ended up waiting an hour and 15 minutes before I was taken in. I am a little idle and motivated sort of person so I can do this but the next person who is super busy might not do it. There are some people for whom these things are really important, and some people will be like I cannot put that much effort into getting this. So, this is why I am saying that if the process of getting prescription could be made smoother, that would make things better because going and buying the drugs is less of a problem.

If expiration on drugs would be longer, everything would be better. We would be wasting less, imposing less on the service provider and making things easier for ourselves too.

Interviewer:

Yeah, I think you have already mentioned the cost. Is there still anything you want to mention about financial barriers in terms of accessing PrEP? It could be a general or personal experience as well.

Participant:

No, I go and for every prescription I pay five hundred rupees and get the drugs. 500 rupees is a fee of some sort, but I don’t know what it is. The drug itself,. u are not really paying for it. 545 rupees are the services charges I pay for the drugs in 6 months. That is not a lot, and most people can do that without an issue. The peripheral tests and consultation, however, are expensive. I think I have also been told to get an HIV test every three months which is expensive and draws blood. I want to get an HIV test once in 6 months or once a year. But three months is excessive for me. I do not want to take it that often. I do not have enough sex to qualify for getting tested every three months. I have a lot of financial liberty and I do not want to assume everybody has that level of financial liberty. I am a small minority in this situation, and I am aware of how expensive all this stuff actually is.

Interviewer:

Are you still on PrEP? Are you using PrEP?

Participant:

Yes, I am.

Interviewer:

Ok. I will be skipping some questions if they are not relevant directly. Coming back to the point which you just mentioned about sexuality and using PrEP in previous response. I just want to inquire about what are your feelings and perceptions with your sexual partners while you are using PrEP. What are you thinking when you are using PrEP as well as connected to your sexual partner?

Participant:

It is a little bit liberating, there is a kind of newness to it. In that moment you also take a bit of comfort, safety and pride. You feel happy and you feel that you have this. There is one less thing to worry about. It actually feels like a shiny new thing. It is something you can take a little bit of pride in. It definitely brings a little relief. Does that answer your question?

Interviewer:

Again, it is open ended. Everything you share is important to us so do not worry that how you responded.

Participant:

I just want to mention one thing, might come later too. I am thinking about it a little, so I am realizing that my recent experiences for the last 6 months or a year, I think only one other person that I have been with was also on PrEP. I do not think any of the other people were also on PrEP at the same time as me that I was with. It sort of really brings PrEP into the conversation. I have been in that situation a long time back when the other person was on PrEP, and I was not on PrEP. I remember that you understand your situation is very different from the other person and your options change a little bit. If both people are not on PrEP, it becomes a very significant thing in the conversation. And if you are with someone who is also on PrEP, nobody wants to talk about anything, it cancels out or becomes irrelevant. But when the other person is not on PrEP, then you understand that their sense of safety is not the same as yours. You are then making the same kind of considerations and making the same kind of allowances, taking care of the other person. Something that is important is whether the other person is on PrEP or not, this unfortunately happens that only very few people are actually on PrEP. Yes, that’s what I wanted to say. Please ask the next question I interrupted you on.

Interviewer:

Thank you so much. You have mentioned a lot of important things that cover a lot. I have a few quick questions and then we are good to go. You have mentioned the experience with pharmacy, clinics, physician and everything. I just wanted to ask are there any other experiences in healthcare while accessing PrEP which may be related to nurses, physicians, students, residents, pharmacy etc. any experience on how they perceive it or when you went to get PrEP, how was your experience and their behavior towards you. Anything specific to the healthcare industry or healthcare professionals?

Participant:

There are a few people, first of all you interact with the person who is handling your appointment which is the receptionist. They have no idea as they treat everyone the same so it is smooth with no problems. Then you are taking into nurses who take your vitals etc, they also do not know what you are here for. If they know because they have my file so they might know, I have never felt like I was given a second glance. I always felt like if I go to an optometrist or a GP, I do not feel any difference. This would have been very different if my specialist was a different person or had a different attitude. I think I really lucked out with a really good infectious disease specialist. I think it is very important for us that we get the consultation without any judgements. I think that is a really remarkable thing to be able to hack that. Then you are at the pharmacy again, nobody has ever looked at me twice or looked surprised or felt like they knew what these drugs were for or anything. It has been super professional throughout. There could be other things but in terms of specifically getting PrEP, I never felt like anyone has looked at me twice, thinking or judging something. None of that at all. Particularly in [tertiary care hospital], this has been very good for me. In terms of students, it has never happened to me. That is because I go to [tertiary care hospital], so I do not know if there are ever students there. I never had students in my room while I was with the doctor. I cannot answer that. Not in terms of Dr ABC’s clinics.

Interviewer:

So, as you mentioned the word judging, I really want to know if have you ever felt stigmatized or judged while you are accessing PrEP?

Participant:

No, not all actually. Surprisingly, because I have always gotten into these things expecting that someone is going to do something or say something, but it has never happened actually. I told you various people that I engage with in order to get PrEP. This also includes the lab where you are actually asking about an HIV test and going to get your blood drawn. That person who is drawing your blood also knows that you are getting an HIV test and kidney function test. No judgement at all ever. Never have someone looked at me twice or was freaked out/alarmed or unhappy to be dealing with me. Never, not even one bit. I can only tell you this specifically for [tertiary care hospital].

Interviewer:

Have you received any social support regarding PrEP access through any NGO, family, friend or any other person? Apart from the people and places you have mentioned yet.

Participant:

I think if I really needed it, friends would have been because I have friends who are doctors but not specifically in terms of PrEP. I do not think so. I chanced upon PrEP because I had gone in fact to Dr ABC for something else. We were having this conversation and he initiated and told me that me that look we have this available. And that is how I got into it. And as soon as this happened, I realized that other people were on PrEP etc. I of course told a lot of people and connected them to the same consultant. That is how this has happened. My source of follow up and primary information in terms that the drugs are available, and I can get them, has been Dr ABC.

Interviewer:

Who is accountable for providing PrEP? Who do you think should be responsible for this?

Participant:

That is a tricky question. Ultimately, I think the government should pay for everything but like I mentioned earlier, I definitely want PrEP to be available for free for people. I am conflicted because there is a part of me that wants to believe that healthcare should be entirely free but there is also a part of me that wants to say that I do not deserve free drugs. I feel like I should pay for my drugs. If I were paying for my PrEP, I would be a lot more comfortable getting it because right now I feel like I am a part of some government program or a sexual minority person and I am told that I qualify for this bit of medical aid so please take this. I am like I do not want medical aid and I want to pay for it. But I do understand that there are administrative concerns. If it is free for everybody then it is. If it is not free for you it also becomes difficult for the systems to make it available for free people who need to get it for free. I understand that. It’s very hard to say that it’s the government’s responsibility to make these things available. I am also thinking do I really want the government to have access to this information. We do not know what is about to happen in this country at this point. We are on these files, and this is not necessarily a comfortable thing considering our country and considering we do not know who has access to this data.

Interviewer:

Thanks for sharing. This brings me to the final question. What else do you recommend or suggest regarding HIV prevention or PrEP availability? Do let us know about any recommendations you have.

Participant:

This is very important. So, a lot of people just do not know about PrEP, I am educated, and I spend a lot of time online, and I watch a lot of television, so I know about it. People generally do not know about PrEP. Unless you really go looking for it, information is not available. There is a serious lack of awareness in general in terms of STIs. PrEP is an advanced level which very few people understand. The most aware are the people working in organizations such as [community NGO]. I think people who have engaged with them actually know a lot more. People who are transgenders for instance or who do sex work seem to have more and earlier access to information or people who have worked with these organizations. Not enough people know about PrEP, and I feel like the information needs to be where people are, for example your dating/hook up applications. Of course, you people are limited, I do not know where you can put this all without making things difficult for people. Information issues and misconceptions are there. Sometimes people think that PrEP prevents all STIs. That’s a really serious misconception people have. There is another problem where people will engage in very unsafe practices because they think that PrEP is covering. There is a whole scope of other things.

Interviewer:

You have responded to a lot of things, but I will rephrase if you think there are other things which come to your mind. Any recommendation or suggestions regarding HIV prevention or PrEP availability?

Participant:

Make it a smoother process to get a prescription and get information out there because it is lacking. I am always surprised and curious as I am always asking people and come to know that PrEP access is actually more than I am aware of. People who are my contemporaries or part of this community do this work independently like sharing information or helping each other etc. But there is not information available openly. The way to reach people is to be where people are and not on television for instance where everyone else is. I do not think I have any other suggestions that I can think of right now.

Interviewer:

Thank you so much for your time and participation in this study. I really appreciate that your contribution would be helping us in bringing some insights into the study. Is there anything you want to ask then I will move to the closure?

Participant:

No, I am all right. There is nothing else.

Interviewer:

I have already mentioned a few things that Dr ABC has already been talking to you. Let me rephrase the important information that you may have missed out. So, the study title is “Exploring the barriers, willingness and preferred ways of using PrEP among men who have sex with men in Karachi, Pakistan”. The point person of this study is Dr ABC. If you want to ask anything and if we want to revert back to you, we will be connecting with Dr ABC. Preet and I are part of the team, and we will be there in the interview part and Dr ABC has already forwarded you a form for E-consent. We would appreciate it if you could sign and send it back to Dr ABC at your convenience.

Participant:

Where will the form come from?

Interviewer:

It will come from Dr ABC. We just need your signature. Please do not mention your name or any personal identifying information. As you know this interview has been recorded, let me assure you that any personal identifying information will be eradicated or removed and will not be used.

Participant:

Thank you, that’s fine.

Interviewer:

If you have any concerns regarding the study, interview or process, you can get in touch with us any time. Your participation in this study is completely voluntary and you have all the rights to withdraw at any time.

Participant:

Sure. Thank you so much.

## Interview 3

Interviewer:

We are doing this study to check barriers and difficulties related to accessing PrEP. This study is headed by Dr ABC. Your participation in this study is completely voluntary and you can choose to withdraw from the study at any point in time. We will send you a consent form which you will sign and send back to us. If you have any questions at any point, you can always stop and ask me.

Participant:

Ok. Alright.

Interviewer:

What are the ways to prevent HIV that you are aware of?

Participant:

There are condoms and there is PrEP.

Interviewer:

Which of the ways are you using currently?

Participant:

I am using both.

Interviewer:

Are there any other ways you have heard of or know about?

Participant:

I did search it on google, and I am not sure but there is a medicine which you inject and then you cannot get HIV. I have heard it from somewhere.

Interviewer:

Alright. These are completely open-ended answers.

Interviewer:

Specifically for PrEP, what exactly do you know about PrEP? How does it work and how do you buy it? What is your knowledge regarding PrEP?

Participant:

What I did was that I went to an NGO and they first tested me for HIV by finger prick test. They asked me to come to [public hospital] the next morning, they took me to [public hospital] and performed HIV and other STDs test. When it came out negative, I went to the doctor, and she gave me PrEP.

Interviewer:

And do you know about PrEP? How does it work and how does it prevent HIV?

Participant:

No, I don’t know that. I just know one thing that it kills the virus as soon as it enters.

Interviewer:

Since how long have you been using PrEP?

Participant:

It’s been 10 days, just started.

Interviewer:

Where are you accessing PrEP from?

Participant:

I got it from [public hospital].

Interviewer:

So far in these 10 days, although it is a very short duration, how has your experience been regarding PrEP? Usage, frequency of intake and general experience.

Participant:

To be honest, I don’t feel any difference. It is not a very heavy medicine, so it is not making me feel any side effects. I just have to eat it once a day. I wake up at 10:30am and I take it.

Interviewer:

So far, your experience has been fine?

Participant:

Yes, it is fine.

Interviewer:

In the last 10 days have you thought that I don’t want to take it or continue with this?

Participant:

No, I have not. Because it is not causing any problems, before PrEP, I once took PEP because I had an experience and I had to take PEP. Luckily the person I did it with came out to be negative so I left PEP. I took for it like one week. It was a very heavy dosage that I started feeling pain in my kidneys. The pain built up slowly and gradually, so I thought I have to leave this. With PrEP, I did not have to face anything like that.

Interviewer:

When you have to access PrEP, how do you commute to get PrEP? Do you get it prescribed or do you purchase it?

Participant:

There are actually two ways of getting PrEP. One was that I went to Dr ABC, he would ask me to get tested and then I would get PrEP from [public hospital] which was the most discrete and safest way. The thing is I cannot tell my parents that I need money for PrEP so I could not do that. Because I don’t earn money yet and whatever I need money for, I need to ask my parents and tell them the reason. That was the safest way and I would prefer that way if I could but I cannot.

The other way is going to an NGO, there are all sorts of people and communities there. You have to face all of them. Even if you do not face them you have to sit in between all of them, which is kind of uncomfortable. They tested me for HIV with a finger prick test and then they called me to [public hospital]. Going to [public hospital] was another experience because there are all sorts of people and then they took me to HIV ward where some people came out to be positive while others were negative. I got very scared by looking at those things but luckily, I was negative, and I got the PEP from there.

Interviewer:

Since you said that you have to get the finance yourself, has cost ever been an issue?

Participant:

Cost is the real issue for me because I do not earn money and I have to ask my parents for that. Cost is the issue but if I get the free option which saves the cost, but your privacy is a little discreet and people start recognizing you a little. You have to face 5-6 people including the doctor, the people who take you for PrEP, and the person who is testing you for PrEP. This happens.

Interviewer:

Since we are already talking about it, this process you have to go through for accessing PrEP, how has your experience generally been with this that might become a problem or currently is a problem that you might leave PrEP because of this?

Participant:

Yes, it is a big problem because I also told a friend that he should get PrEP but he said that he was scared of going to NGO and facing all sorts of people and then into the hospital and facing the situation there. Even I was very scared doing that. Even now, I am always worrying that next month I have to go so how will I go? So, I had put on my mask and wore a cap because I was trying to be as discreet as possible, but I had to give my CNIC there and everybody was getting to know your name which for me felt a little uncomfortable. But if I could afford it, I would rather opt for the [public hospital] Hospital option because going to Dr ABC, telling everything just to one person and then getting tested to get the PrEP from the medical store downstairs. So, this is the problem.

Interviewer:

So currently you know that NGO has it available and for [public hospital] and [public hospital] you are telling that you access it from there, right? Apart from this, are you aware of the availability of PrEP elsewhere? Where is it available and what are the ways to access it?

Participant:

Yes, there are other places where it is available. I know this. I do not exactly remember the names of all these places but there is one hospital in [another part of the city] where it is available somebody told me. But the place is such that it has different kinds of people, so I have never been there. Even if I go there, they will test me one day and then the next day they will call me again. An entire day waiting is required there so I was not willing to do it. In [public hospital], you get the results within 1 hour.

Interviewer:

And the repeated travel, you thought was also an issue and you said that I don’t want to go again.

Participant:

Travel was not the issue. The real problem was going there, I had already experienced one thing and then I had to experience it again. Back-to-back in two days.

Interviewer:

So, the repeated exposure to people and the questions was what you did not want to do?

Participant:

Yes, yes.

Interviewer:

Right now, the experience you are going through while accessing PrEP, are you satisfied with it?

Participant:

I am actually not satisfied with it. I actually prefer, that for PrEP, there should be a different place because those people take PrEP who do not have anything diagnosed. They just take it as a precaution. I think PrEP should be available in NGOs because if you go to NGOs, there you do not have to meet that many people but going to the hospital is a different story so I believe there should be a separate place for PrEP. Or someone like Dr ABC who is more affordable. Like NGOs are free, there should be doctors who are fee to whom you can go in a discreet manner while securing your privacy, to whom you tell the entire story, and they get you PrEP from the same hospital at the same day, so you do not have to face all those people. So, I am not satisfied.

Interviewer:

So according to you that would be the ideal way of getting PrEP so that everything is done in the same place.

Participant:

Yes, all the work is done in one place, and you do not have to face a lot of people. Because I personally know a lot of people who are scared of doing all that. Even though I am scared, and I put in a lot of courage to go there, otherwise I would have never gone. I was very scared. Otherwise, I would have started PrEP a long time ago.

Interviewer:

Definitely. Understandable. So, till now, whatever your experience has been, especially with healthcare professionals regarding accessing PrEP, has it ever happened in the experience that you have faced judgement or generally while accessing PrEP, how has your experience been with health professionals?

Participant:

For NGO, I did not feel any judgement. In NGO, I was feeling a little bit more open about it because all the doctors there were very kind and chill with it. The [public hospital] on the other hand had different kinds of people, there were a lot of benches and families present. So, I was worried what they would be thinking is that I have the disease, the tests that were being done, the doctors I was talking to who were giving me PrEP. If it is a male then I am fine, if I have to be very honest, I am bisexual that is the reason I am getting PrEP. With a male who is already bisexual or gay, I can be very open with them, and I will not be afraid in front of them so I can talk openly but if it is a female, I will feel a little uncomfortable. There was a lady doctor there and I had to face three ladies. First, I got tested by a male, when test result came out, I went inside to a lady who asked me questions regarding last exposure, and she sent me to another room where I asked a lady that I need PrEP. She then sent me to a counsellor who signed the paper, and she sent me back to another room where I finally got the PrEP. I had to face a lot of people; this is the only thing I was afraid of.

Interviewer:

Ok. Because you faced a lot of people in this situation which was a problem. Were the concerns regarding your confidentiality and safety?

Participant:

Yes, yes. It was all about confidentiality.

Interviewer:

Are there any specific concerns regarding confidentiality and safety that you want to talk about?

Participant:

Obviously, we are living in Pakistan where being bisexual or gay is a big taboo. You cannot just tell it to anybody. You are always worried that nobody should be seeing me there. Even if someone is already involved in these things, they also do not get to know me. If people are into these things, they will act as homophobic so that people think they are not into these things. Most homophobic people are those who are most into these things and that is what I have gathered till now. I am generally scared that nobody looks at me, nobody knows about me, I do not face a lot of people and I just talk to one person and the work is done. That is what I think is ideal.

Interviewer:

Do you think these concerns at any point will hinder your process of accessing PrEP or your willingness to access PrEP?

Participant:

Yes, it will. So far, I have not found anyone in those places but if some day I find someone I know, I will not go there next time.

Interviewer:

Ok, it will result in you never going back there.

Participant:

If I find someone I know, I will never go back since it is a privacy concern then.

Interviewer:

Before accessing PrEP, was there any professional you thought you should have connected to for any information on PrEP or services? Or you thought there should be a helpline or professional who I could call and ask regarding accessing PrEP?

Participant:

There was no helpline as such but there was a doctor in the NGO with whom I talked in detail, and he recommended me then.

Interviewer:

Do you want that there could have been such a resource where you could have contacted before?

Participant:

Yes, there should have been a resource and there should be a helpline talking about the possibility of risk factors and what the risk factors are. Sometimes, you do not have risks with some things but since you are new to these things that you are actually afraid about it. There should be a PrEP helpline and one for daily concern sharing too. Anything unusual or doubtful comes to your mind, you can share with that person who can validate your concerns as being right or wrong.

Interviewer:

Right. Now that you need information at any time, do you have the resources you can use to receive that information?

Participant:

Right now, I have the contact number of the doctor at the NGO. Whenever I am overthinking about anything and whenever I have questions regarding the use of PrEP, I always text him and he guides me. I told him that I wanted to use need based but it was like taking two pills, then 1 pill after two days and so on. There were chances of mistakes with this option, and I was afraid if I could take so many tablets all at once or not. I am a very lean person with [weight withheld], so I am worried that it is not a very heavy dosage. I asked him and he told me that I should take it daily. I forgot to take my pill at 10:30am and I asked him so he told me that you can take the pill within 12 hours, as soon as I came home at 5-6pm, I took it. There should be a person who is easily accessible if my friend needs it so I can quickly give his contact number to my friend, and he can talk to him. This resource should be there.

Interviewer:

Right, thank you. So, are you receiving any sort of social support from friends, family or communities?

Participant:

Not from friends and family but community because I have made friends in the community.

Interviewer:

And you told me you are accessing PrEP in tablet form?

Participant:

Tablet form, yes.

Interviewer:

Do you think if there was any other form in which PrEP was available, would it have been better? Or other forms of PrEP should be available such as injectables or anything else?

Participant:

Yes, this can happen. If injectables are available and you have to get injected once a month. That would be good too because doing it once a month would do the job. Because the issue with PrEP is that you have to hide it somewhere in the drawer and you are scared that nobody will see it. I can keep it in my cupboard, but some people do not have the privacy to do that. For some people, their mothers or someone lese sets the cupboard so they can accidentally find these pills. Injectables would be a good option if injected once a month and then you have to go the next month and not eat it daily.

Interviewer:

So, do you think that would be preferable over the tablet form?

Participant:

Yes, for me at least.

Interviewer:

Are there any difficulties or difficult perceptions you have faced while being on PrEP when you are connecting with your partner?

Participant:

No, actually people are very okay with it. Even before when I was not on PrEP, I would feel very safe doing stuff with somebody who was on PrEP. Here people do believe that PrEP is the safest option, even safer than condom. I do not believe that condoms give you that much safety because I was always afraid and could not even do proper penetrative sex. I started doing it after I got PrEP. I wanted to try it and that is why I got PrEP otherwise, I would not have done it. I would never have done it with condoms as I believe that condoms do not provide that much safety. It can tear or break anytime so there is too much pressure on your mind.

Interviewer:

Ok. While being on PrEP and while accessing PrEP in the short duration that you have been taking it, have you ever faced any difficult incident or stigmatization? Anything you want to share?

Participant:

No, because the only people who know that I am taking PrEP are the people that I know from the community otherwise I have never told anything to other people straightly and neither will I tell them that I am taking PrEP. because of course there is the stigma, and they will judge me for that.

Interviewer:

Right. In your opinion, who should be responsible for the provision and dissemination of PrEP?

Participant:

This the primary responsibility of the government and I think they are doing that. But I believe that it should be dedicated rather than having a ward in the hospital. PrEP should be even separate from the HIV ward so people can go there easily and be discreet about it.

Interviewer:

Ok. Anything else you want to recommend or suggest regarding PrEP availability, accessibility, cost issues? Any suggestions you would like to make which would make PrEP accessibility much easier for you as well as the community?

Participant:

I do not think that home testing would be fine for everyone because not everybody lives alone but I do believe that some people live alone, and they may fear going to these hospitals to get PrEP. So, if there was a way that they could get PrEP at home just like [a diagnostic lab] comes to your home to do tests, similarly this system could be there.

Interviewer:

You mean it is delivered at home?

Participant:

No, like [a diagnostic lab] comes to your home to take blood samples for testing like during Covid.

Interviewer:

So, you are trying to say if the medication assessment can be done at home?

Participant:

Yes, the medication assessment is done at home and the next day medication is also delivered at home.

Interviewer:

Ok. Are you saying this with regard to privacy, that if it is done at home, there will be no contact with other people?

Participant:

Yes, there will be no contact with other people. There is an issue with this for people like me who do not live alone but those who live alone at home, it is possible and easier for them.

Interviewer:

These are all the questions I have. If you have any questions for me, I would be happy to answer.

Participant:

No, thank you.

Interviewer:

Thank you so much for participating in our study. As I mentioned, your participation is completely voluntary. Any of your identifying information will not be used in the data or analysis of the study. Dr ABC will forward you the consent form, if you could sign it and send it back to him that would be great.

Participant:

Ok, I just have this one question that you are recording this session, right? Even if my identity is not revealed but my voice will be.

Interviewer:

Yes, it is being recorded. So no, it will be just me and one member from the team who will transcribe this (convert to text). Apart from this, this recording will not go anywhere, and nobody will listen to it. Your voice will not be made public at all.in text, there will be no identifying information, no names, no number nothing at all.

Participant:

Ok, thank you.

Interviewer:

Thank you very much for your time

## Interview 4

Interviewer:

Thank you so much for joining. Would you prefer we continue in English or will Urdu be a preferable language?

Participant:

I think English, Urdu, both. I'm okay with both.

Interviewer:

Okay, so to introduce the study, we're conducting a study to understand the perceptions and usage of PrEP. And I am one of the study coordinators and along with our principal investigator, Dr ABC, who I'm sure has already talked to you a little bit about it. And you know participation is completely voluntary, and you can choose to not be a part of it or leave the study at any point you want.

If it gets uncomfortable at any point, please let me know and we will stop the interview. Also, the interview will be recorded, but it will not be shared anywhere. It will just be used to transcribe whatever we talk about. Then when we analyze the data, we'll just use the text and any identifiers, your name or anything that could possibly identify you will not be present or mentioned in the data at all.

Participant:

Got it.

Interviewer:

Okay, perfect. So ready to start?

Participant:

Yep, lovely.

Interviewer:

So as an opening question, could you please tell us what you know about general HIV prevention?

Participant:

I think in the past two years, since we've started having PrEP, that is only when the conversation started. Before that, there was absolutely no conversation, even amongst well-read communities of, you know, MSM men. There was no conversation about it at all, because of the healthcare system, the general conversation around it is so minimal, basically next to none, actually.

There's no concept to begin with. If there's something that needs to be done about it, or what are the methods and ways, because of course the taboo that comes with it. So very naturally there is no conversation or concern around it. But then just because having friends who work in healthcare, there was a bit of a thing going around like “There is something called PrEP that is available publicly and could be consumed and would prevent you from HIV”.

But that was very recent, that was also something that happened only because there was a dear close friend, who happens to work in healthcare. But otherwise, even till now, the best of us, the most, those who even have the access to the best of healthcare have absolutely no idea, have no interest also at the same time, and some kind of reluctancy about the whole situation surrounding PrEP or the concept of HIV, because it's been brushed under the rug so much and has been centered around certain activities. The idea of getting infected is very foreign as if we are immune to it or it would never happen to us, whereas it could happen to the best of us. It's just like any other thing. It's like diabetes or blood pressure which you would normally go and get it treated because the conversation around those illness or, those diseases is much more or bigger than it is around HIV.

The only information that has been given about how big it is is because I personally read it too, but otherwise if I look around me and I do speak for them, not on behalf of them. There is zero willingness which is stemmed by massive reluctancy of going through the process of obtaining PrEP. In a nutshell, that is my answer to “What is your understanding of HIV?” There is none.

There's just a perception of it that it is something that people who, like sex workers would have or if you're somewhere, not in Pakistan, but if you're somewhere, let's say abroad, there is a higher risk of it. That's the perception around HIV.

Interviewer:

Okay, thank you for saying that. Since there's very little information about PrEP, is there information about other modalities of HIV prevention?

Participant:

Very recently, that was also, again because I like to read into things. But if I take away that experience, no, there has been none. I know it, but otherwise, I don't think. People haven't even gotten to the idea of PrEP.

And it was very recent that there was a fellow friend who had a scare of being contracted, with HIV, or had sex with a partner who was active or something. That is when the conversation began of obtaining PrEP. But that was also in itself a big hassle.

I don't know how easily available is Plan B that normally a female would consume to prevent pregnancy. But that's when the conversation started around PrEP. It was that it needs to be obtained in the same method, which is a long process and a bit difficult, tedious one. But to answer your question, personally now I know that yes, there's PrEP.

But if you ask me holistically, there is very little to no awareness about that. There is so much less information about PrEP, it has not even reached PrEP yet. So, there's no awareness around the preventions or the methods to prevent HIV.

Interviewer:

I'm talking about you, personally. What steps do you take for HIV prevention?

Participant:

All right, so basically, I think as soon as I started reading into that there is something called PrEP and coming to Pakistan, that is available. It works this way, that it does prevent you from contracting HIV. Because I happen to have access to good healthcare. I have access to a private facility where I can go obtain it.

And then, do you want me to give you these lengthy answers of how I got there and how it happened? Or do you want me to give you a very brief answer? Because I feel like I'm kind of giving you a whole rundown.

Interviewer:

These are very open-ended questions. Whatever information you think is necessary, please go ahead.

Participant:

All right, could you repeat your question again, please?

Interviewer:

Yeah, so what steps do you take for HIV prevention?

Participant:

What steps do I take? When PrEP came into Pakistan and there was an access/basically a route to it that where you can go and take it and have it, which would make you feel safe. That's what I did. I went and I got myself PrEP, the simplest way of protecting yourself against HIV, which is PrEP.

Interviewer:

Is there anything else you're using for preventing condoms or anything else?

Participant:

Oh yeah, of course. Condoms, yes, of course, 100%. You've got your condoms, but of course, as we all know that condoms are not safe and have a chance of slipping or tearing or XYZ, many other things. Sometimes if not being available or sometimes let's say when you are with a partner that you trust, that's when you don't. And then that's where the PrEP comes into play. Condoms and PrEP. The usual.

Interviewer:

And what do you know about how PrEP works?

Participant:

Could you repeat that again, please?

Interviewer:

Yeah, do you know how PrEP works? Do you have any information about how PrEP actually works?

Participant:

I do, I do. So, there are two methods of using it. One is when you have unplanned sex, you don't know when you're going to have it, when you're active, actively active. There's a 30-day consumption situation, which I'm not too sure about how that works because I don't fall into that category. I fall more into the planned category situation. And I know how that works.

If you go online, you have it available. If you go to your healthcare worker, they also have the information they provide you with, which is you consume two tablets two hours before or 24 hours before, and then you follow it up with one for the next two days, after consuming the first two tablets.

Interviewer:

Okay, okay. And generally, what has your experience with PrEP been like? Has it been a comfortable experience? Or has it just generally been an experience that has not been super pleasant?

Participant:

Experience with?

Interviewer:

With using PrEP in general?

Participant:

In general, I think it has some side effects. It does, which is basically the stomach being upset. It makes you a little queasy. It's because it's a heavy tablet. You can tell even by just consuming it or even just holding that tablet in your hand, you can tell it's a heavy tablet and it does have a bit of an effect. Sometimes it does not, but usually if you do take it, it gives you this queasy feeling and sometimes ends up with an upset stomach, perhaps.

Yes, so that's just been the experience. But apart from that, I mean, just to have something that will make you safe is in itself a blessing. So, you know, that's been the experience, basically.

Interviewer:

And has that, along with any other reason, made you feel like you should stop being on PrEP?

Participant:

No, not at all, actually. I think I fall under one of those who actively advocates for it, because I think the, you know how COVID happened and everybody would say, oh, it's just in China. It's just in China, but then it came, it spread all over the world.

Same goes with HIV. I think Pakistani people have the notion that, oh, this is not in Pakistan, or if, this is for straight people These are the notions in Pakistan usually, people assume that this is something for dirty people, I would say, they consider that. It's never going to come to us, whereas that's not the truth, you know, that's just weird things that people have made up along the way in Pakistan to keep their sanity, to make up these lies just to make yourself feel better. It's one of those situations. Whereas I don't, and I am one of those who actively vouch for it, recommends it to the friends, let them know that, hey, guys, there's something out there that will make us feel safe but also actually keep us safe. So that's my take on that.

Interviewer:

Wonderful. How long have you used PrEP for?

Participant:

It was last year, so it's been one year, but funny enough, kind of sad also actually, given the inactive time. I think consuming PrEP was more about having something on hand that will make me feel safe. It's been one year, but I've only consumed it on a couple of occasions. That's it.

Interviewer:

Okay. And where do you purchase it from, get it from?

Participant:

I got it from [tertiary care hospital]. That's where I get it from. I go see a doctor, and then they run some tests and hand me the form.

Interviewer:

Okay. And has the commute been difficult yet, or has the cost of it ever been a problem for you?

Participant:

Now, okay, so for me, it hasn't been a problem because I understand that this is something that is health related. There's a certain amount of money that will go into it because, again, that's what you do if you break a leg or if you want to go see a doctor for any kind of flu. But that's me.

But if I turn around, look around me, and I'm doing that thing where I'm speaking for the community a little bit over here, there have been a few issues about, A, locating it, B, regarding the money around it, because, of course, it's a private hospital. It has a certain amount of appointment money. Then there's a certain amount of money that goes for the tests. Then you also pay a little amount to get the PrEP. So yeah, those are some of the issues honestly. If you ask me, if I knew that prep is kind of one of those things where I go, I am already on the list. I have seen a doctor. I've done all of those things. And then there's a simple process of consuming this certain medicine that I just need to do these tests that the lab will do it for me for a certain amount of money, like 500 rupees or something. And then you just pick up your meds and you're good to go, right? But that's not the case. That's not the case.

And that should be the case for the majority of the people who want to consume it because to book an appointment for 5,000 rupees, you need to have a certain amount in your pocket, which I am fortunate enough to have. But if I look around me, the [young adults] or active boys or moving on, even those, let's say, regardless of their age, but this is something that they are not, A, willing to spend, B, oftentimes do not have the means to spend this kind of money. Does that make sense?

Interviewer:

Absolutely. So, are you personally satisfied with the process you have to go through to access PrEP?

Participant:

I am. I very, very much am. Because it's done with a lot of sensitivity. It's done with a lot of care, very nicely, very sweetly. So, I am. But I have a question.

You know, these questions that you're asking me, and because I'm a part of the community and I've had these conversations, the same conversations, the questions that you're asking me, you know, with a lot of other people. And so, should I be answering holistically or do you want me to keep answers just based off of my experience only and only?

Interviewer:

Both, actually. Both will be very helpful. Your personal experience will be very helpful, but because you're a part of a community and you have access to their perception as well, from conversations with them, also being around the people that you know, if you can give some insight that is more holistic and representative of their view as well, that would be very helpful as well.

Participant:

You know why? Because I actively advocate for PrEP to all my friends. And I speak to them about it because, so you see more than preventing, it is something that helps you avoid that day when you're scared, that day when you end up in a situation. I've gone around and I've spoken to a lot of people about this. And so, I know the answer, some of the replies that I've gotten, some of the things that they've said, which have led me to have a better understanding of all. So, this is my experience, but X, Y, Z, who fall into different categories or have different pockets or different experiences or different kind of personalities for that matter, have a very different approach to getting PrEP.

Interviewer:

Right, I'm just adding a little bit. So, about the availability of PrEP, you access it from [tertiary care hospital], but are you aware of any other access points that are being used to get PrEP from?

Participant:

I do know that there's PrEP available, through government, government hospitals. But before I even get there, there is such a bad reputation regarding government hospitals and then let alone to go get a medicine which is for gay men or men who have sex with men. So just the idea of going in for that at a government hospital is very unnerving.

Interviewer:

Hmm. Okay, so in your opinion, what do you think would be the ideal way of getting PrEP that would be most accessible not only to you, but generally to the community as a whole?

Participant:

Sorry, could you repeat that?

Interviewer:

Of course. In your opinion, what do you think would be the ideal way of accessing PrEP?

Participant:

Okay, so the ideal way of accessing PrEP is, first of all, I think awareness because the most important thing is, let's say we open up the most convenient space to give out PrEP or where people can have the easiest access is awareness. If you walk into a hospital, you will never know in a queue if there's PrEP available unless you've heard it from someone, unless someone has mentioned it to you one-on-one personally. Or even, for example, let's say you search PrEP Pakistan, you will just find United Nations or UNO, that there's PrEP being launched. That's fine. But there are no simple steps or measures that you can take like “All right, I need PrEP. What do I do?”

PrEP, Pakistan, where? So, then you should be out there simple with a certain something written that, hey, is PrEP available, blah, blah, blah, this and this, book an appointment, something that makes you feel a little comfortable that, all right, we've got this person over here doing this. So that's, that's, for starters.

Then at the same time, if I say [tertiary care hospital], now the thing with [tertiary care hospital] is that that's a private institution which is costing money, right? Another easier way to get it is, and the more comfortable way, because these are kind of things that where you need comfort, where you need to be comfortable that there will be discretion because there's already so much guilt and shame around in this country on being sexual, let alone to go in a public hospital and get it. So, one of the things that I don't know how to put it, but in the longer run, the idea, the bigger idea would be to have a clinic but that must be in a spot or in a space, which needs to be smartly placed where everybody can kind of go, you know.

Interviewer:

Location-wise?

Participant:

Sorry, what?

Interviewer:

Smartly placed location-wise, geographically?

Participant:

Yes, geographically. Sorry, lack of a better word. Yeah, geographically, because where people go to [tertiary care hospital], it's a big hospital, it's nicely done and everything. I want to get my own national medical hospital for that matter, but I'm talking about [a large city in the country]. It could be other hospitals, otherwise. But for example, for flu, for knee injury or anything you know where to go. So, if you take a step back, people don't even know where to go and that's the big, big problem. They don't know how to do it on their own. So, no matter how many times you go talk to a person or a friend and you recommend and you kind of try to do it, I don't think people are going to do that unless there's an awareness for it, there's a marketing for it, there's an access to it, which flows easily.

Interviewer:

Makes sense. And do you think you or the community would have liked to connect with specific people or professionals to have more information regarding PrEP? Like a point person who you could call, text, email for information.

Participant:

Yeah, yeah, like there needs to be a helpline. There needs to be a number that, for example, let's say I have, because since I'm somebody who talks about it a lot more to my friends and people, and I've had a personal experience of going and getting it. But that shouldn't be the case because I'm not that person. It should be an officially appointed person doing what I am doing for a bunch of my friends instead. Who's doing it for everyone in the city?

This should be on [tertiary care hospital]'s website, there should be PrEP contact or PrEP related appointments, and a number given, like a UN number, or you can call because nobody knows. It was also through a person that I found out that PrEP is available at this hospital. Otherwise, I would never know, even if I call the hospital helpline or hotline or try to make an appointment, but I will never know. I will never find out, if I tell them PrEP, where is it available, where is it, what is it? They will not, I don't know if that's going to work. The most important thing that needs to be done is that there needs to be a number. For example, if we're talking about methods, there are two methods. If we go to a private village, if we're talking about [tertiary care hospital], then [tertiary care hospital] needs to have it up on their website. It needs to show up when you write PrEP Pakistan.

It needs to be one of those things where you walk in and if you ask the pharmacy for PrEP, so they should be able to give it to you. Even if the doctors are not available, the information regarding PrEP, and this is an actual incident which happened during the weekend of a holiday. It was a three-day weekend and a friend was in desperate need of PrEP, but there was no way to source it in a way where you can just walk up to a pharmacy and be like, all right, I need PrEP till at least this weekend ends, I can be safe for the first two days and I can consume PrEP till the doctor opens up on Tuesday because there's a window and that window could have been easily escaped. But that wasn't the case. There were all these calls that were being made, links to find who can get the PrEP, who can source it, but that was our privilege, but what about those who don't have it? Where do they go, who do they call or who do they go up to?

Interviewer:

Right, so what I'm understanding is that this goes on through word of mouth from friend to friend and a person. There's no official established system currently working, right?

Participant:

Yes, there's no established system. It is a small example like I recommended somebody a restaurant so they get there and eat but the restaurant is somewhere in between twenty thousand other offices hidden and nobody knows about it and what goes inside. There is even food being served there and till this point putting food pictures on their Instagram, and then, all right, here's the link. You give them the link and they can sit with it and they can scroll around it. They can see all the information that comes out of it on a daily basis.

Then that does the work to pull the people in who want to come and get the food. If you flip it, I'm talking about PrEP in that way. I'm using this food reference because of my professional background. But if I change it, I say I'm in desperate need of PrEP. Doctors are not opening up. I don't have an appointment first of all. Another thing, no emergency appointments so you have to make an appointment through a proper channel and this and that, but for that also, you have to wait another two days. So that's 48 hours past then another day where you have to make an appointment and land up on that appointment and then get tested and then get the PrEP, you see how 72 hours went to gone for the person who is supposed to feel safe or supposed to be safe has no means to go somewhere and be like, at least till I go and start solving these things out or sorting it out, which is to get the appointment or see the doctor or run the test at least in that meantime, I know that this is that spot where I can go and get my two tablets of PrEP just for those two days that I can feel safe. Or even let's say this person is traveling and is unable to order that and you don't know if that doctor, or that person is going to be present or absent or is out of country, could not be available. Right? These two, three appointed sources. What if they're not available? There's no channel so we can say at least for this one week, I've got my meds, I'm sorted. I'm safe. At least I'm out of danger and then not at all.

No helpline that one could call and say, listen, I am in dire need of PrEP. They respond, “PrEP? You have your time” and whatnot. But if you don't want that, and this is also based on the personal experience that I went through, that my friend went through, actually there was a big issue like, “Oh, how do we get it? Because it's a public holiday for the next three days. And how do we make it work? How do we move forward?” Because the whole point in that moment is to be out of danger because you're in a window of 72 hours. Does that make sense?

Interviewer:

It makes sense. Okay. Thank you for sharing that. While we're talking about emergency situations, very tense situations, do you receive any social support regarding PrEP and accessing PrEP in general?

Participant:

Would you repeat that again, please?

Interviewer:

Um, do you, do you have access to social support when it comes to talking about PrEP or accessing PrEP from friends, family, or communities?

Participant:

Yes, of course. Because social support is a community-based support more than family support. Friends, of course, it's just gay people. People think that it's not a straight conversation. It's not, so it's more of a community-based conversation. That also kind of comes into play when there's a willingness to listen. So, if you talk about support, there is no conversation around it as much to even gather support to begin with or to even feel supported. It's more like something that you're basically doing on your own. The idea of just getting PrEP to people is horrifying. It's so daunting, but even if you have the money, the means, and just the idea of doing something like that will make it obvious. Because the thing is the method of going out and getting it is basically you are outing yourself in a way that you're putting it in the system. And it's so daunting. It's so, so daunting to everyone. I remember my first appointment and I was so scared, because you see, it has to do with the country that we live in. It has to do a lot with the way we've been brought up. It has to do a lot with our thinking and the way we think about this kind of life. So, you're going out in public to do something which has been shunned, which is something dirty, which is something hidden. So now you're going out there in public and get something at a hospital or whatever it is. Believe me, the number of times everybody has asked me.

Interviewer:

Okay. Okay.

Participant:

Either way, just the idea to begin with is just so scary and it's so heavy that they would disregard it.

They would rather be not unsafe than being safe because there's a little hiccup in between. Because imagine the generational fear back in one person. And then when give them something, even when it's for their safety, but there are some bits of pieces of being out or lack of privacy, or you at least sharing personal information coming out that they would prefer to not go through that and stay unsafe. Does that make sense?

Interviewer:

Right. Absolutely. So, have there been concerns regarding confidentiality and safety while you're in the hospital?

Participant:

Not in the hospital, but of course there is a general idea or thought in the head that, oh, all right.

Confidentiality. How do we make it? How do we make sure that it's going to stay intact? Because of course there's a whole list of information that has been taken down, from your father's name to your mother's name, to you, where do you live, what you do, everything. Basically, what a basic hospital would do if there was any other case, so when you're doing that, that kind of puts you in a spot where you're like “Oh, I'm going down in the system as one of the PrEP consumers.” which obviously leads to the thought that I am a homo or, so how do I tackle that? How do I feel safe in that area? Because that is the thing that everybody either say, I thought shuns the idea, but they don't shun it, but I would say they're scared of it because it's like this little big bridge that you have to jump to get to the other side of safety. And the bridge is so scary and has been made scary over the years, because of the country that we live in, the people that we are surrounded by, they would rather not cross that bridge. Or either they would look around and say “Hey, would you accompany me through this? Would you help me walk through the bridge?” But that's a friend, right? There should be somebody else who does that for those who are not friends, but those who want to get through the bridge, but don't have the brand to get them through this, then comes the confidentiality bridge.

It's only after you've crossed the bridge, it's only after you've made up your mind about them getting there. So, when you cross the bridges, when you do the entries, that's where you were like, “Oh, how do I stay anonymous? Because my entire life has been about staying anonymous” And now here we are giving away all the information, potentially putting us in a spot where this is private information. How do we make sure that stays there? Because then you're in the system.

Interviewer:

Right. And has that concern, that thought of being in the system officially ever made you step away from accessing PrEP? You know the concerns are too big versus the benefit of getting PrEP or the whole process of officially registering, getting the appointment, getting tested. Do you think that makes people want to not go through, with an access to PrEP ultimately?

Participant:

Yes. A hundred percent. I have actually gone through that process once, so I have a familiarity with it. So, I will do the same again. But there is also a reluctance.

Still, even after doing it once, there is still a reluctance. Why? Because there is a process in this as well, which is of running two tests, booking and getting an appointment. It's minimized for me, but we'll ask some questions, we'll do it. That's fine.

I understand. But for those who have reluctance, a hundred and ten percent, yes, they would not.

Interviewer:

And what your experiences generally have been with healthcare professionals, whether they're accessing stuff, you know, nurses, residents, maybe even medical students, pharmacists?

Participant:

So, I've heard a few things about the public hospitals, because of course they give you a look and that's all they can do, but there's a look. Otherwise, private hospitals know, because I feel like they kind of know that if this person has showed up and is over here as well-spoken or well read, then I just feel like there's generally a better experience. I'm just comparing the, the public and the private over here at this moment right now.

So, the public it's been, okay? It's been actually good to be honest, in my experience. But if you look at the public one, it's a bit press insensitive a little bit, which is exactly why there should be none. It shouldn't be public. It shouldn't be private. It should be a spot altogether separate because I'll give you an example in relationship to this. How you have these pregnancy units or some separate situations for pregnant women, there are certain small clinics also, which are just based off of pregnant women, because all the women can show up there in their privacy with no men, where everything is about having a baby and centered around it. And then I don't want the small, small clinics. I'm going to talk about the big private hospitals. And I think that is the kind of approach we need for PrEP. And then followed by STDs and so on.

Interviewer:

And have you ever felt stigmatized while you're accepting PrEP? Has there been unpleasant incidents or anything at all while in the process of accepting PrEP?

Participant:

While getting PrEP, you mean, or generally?

Interviewer:

We can talk about both if you'd like.

Participant:

Generally, I mean, look at us where we live. So of course that's been a daily experience. Yeah, 110%. But if you talk about obtaining PrEP, I told you that if I do not enter the doctor's room or if I'm not actually picking it up, nobody really knows and nobody really bothers because it's a bit faster but that's that. But if otherwise no, while obtaining it, no. Stigmatized to me, it's a heavy word. But if you look at it in general, the stigmatized process of our general life is so much that there is already a built-up perception of, okay, if we enter this building or if we go see a doctor, we're going to get stigmatized. We're going to get the look; we're going to be judged. So, a lot of that nurture seeps into the process of obtaining PrEP.

Interviewer:

Right. And what are the perceptions about accessing PrEP with regards to your partner who may or may not be accessing PrEP as well at that time?

Participant:

Could you elaborate on the question?

Interviewer:

Of course. So, has there been a different perception about being connected to them? Does it sort of boil down to, or bring in question, your comfort or trust with them when the one of you is on PrEP and the other might not be, has there been questions about it or any difficulty being connected to them while you're using PrEP? Do you have to explain yourself that, or I'm using this to protect myself or anything at all?

Participant:

Oh, like that. Oh yeah.

Interviewer:

Yeah. So like, does it become a barrier staying connected with your partner?

Participant:

No, I mean, I think it's such an individual experience that I can only share mine. So, it's about what you can do right for yourself and how you can do right by yourself? It starts there. So very naturally the other person then has to say that you can talk about it and then the partner either needs to hold their end of the responsibility and be thoughtful and be forthcoming about it. So good. But if not, then at least you are the one who's taking measures to be safe, at least, that you are safe.

You've spoken about it. You've shared your parts in it, on why you are taking it on and what's the purpose of it, because it is to feel safe. And if your partner has a reluctancy, not taking it himself, that's on him, but to stop you also from taking it, it's almost also in other words, saying that I don't want you to be safe because that's what it's doing.

It's making me feel safe and it's keeping me safe. So, if you have any objections around it then I can help you understand it better, explain to you the why's of having PrEP on me or taking it also. But if there's objection around it, then potentially what you're saying is that you have a problem with me being safe. You're taking away my safety potentially by showing any kind of objection.

Interviewer:

Right. Okay. So, I think you mentioned PrEP as an oral tablet. Do you think that is the most preferred form or would you like PrEP to be available in another form, injectables or anything else that you feel would be more preferable to you?

Participant:

I mean, I don't know what it's called. Is it called IUC or something that goes up in the arm of women that they put to prevent pregnancy? So, something like that would definitely be appreciated, even though I feel like it would be a bigger task to convince people, because let everybody know, we haven't even scratched the surface with PrEP as a tablet yet, so injectables also is again, one of those things, which is, you have to go to a doctor to get it done. Unless it's something that is going to last you for a month or two months or so and so on or has a longer lasting. But I think we've already come up with tablets, it is already such an easy and convenient solution that whatever comes after this must only have one thing, which is a longer lasting effect.

Interviewer:

Like you said, injectables, oh, all right.

Participant:

So, if there's a PrEP injectable, then it probably needs to last for like three months, yeah. That's the feedback on if there's any other method that where people are looking into making PrEP, then it has to be done in a way, which is like, all right, get your shots every six months or three months and you're good. That would be preferable, because that would also mean no hospital all that often and stuff. Yeah, that would kind of take away a lot of things because then people are just coming in for the shot and it's like a PrEP shot that you come in on an emergency room and you get it.

But I understand that you also have to do some tests early hand, you check if you have your kidneys working fine and other stuff. If there's an injectable that comes in or one of those things that I don't think men would actually take up that thing up their arm, but yeah, if there's an injectable that keeps you safe for six months, that's it because that's how long also the PrEP tablets last. I think six months or one year of expiry. So, that I think it's like just COVID vaccine, you get one in six months and then I'm good.

Interviewer:

And in your opinion, of all the changes and all the provisions we've talked about, who do you think should be responsible for the general access and information for PrEP?

Participant:

Could you elaborate your question?

Interviewer:

Of course. So, all the changes and the things that we've talked about, information on it, about locations and clinics for it and generally PrEP without having to pay so much money for it. Who do you think should be responsible for creating all these opportunities and services in Pakistan or generally?

Participant:

Okay, so there are two ways. Of course, the government won't step up because I mean, there are people that are dying, so they don't give two shits about it. Or if they could, that would be lovely. If they could step up a little bit. They've stepped up, just the fact that there's PrEP available in Pakistan itself is such a big move. So, I think they've done the bit that they could. But if I am talking about it, because I've obtained it from a private institution, then I'm looking forward to perhaps having smaller things. First of all, easier access.

Who do I call? Where do I call? A FAQ? I mean, literally a cell phone or any small thing would have an FAQ page. What is PrEP? Where can I get it? If I have a hospital in the area, they should have a PrEP page. That's it. It should not say MSM, this, that, nothing. It's just PrEP and PrEP's FAQs. To get your PrEP, call us now or book an appointment online.

I feel if the smallest step could lead to bigger change and then we can talk about what to do next. But if you just type PrEP Pakistan in Google, all you get is that PrEP is in Pakistan. Where is it? Who has it? No one knows.

Interviewer:

Okay. So, both of them, like we're thinking a more hybrid approach.

Participant:

Yes. The women should do their part and private institutions that are actually making it available should do their part to make it available.

Interviewer:

Right. Yeah, Okay. Lovely. So, we've talked about a lot of recommendations and suggestions. Is there anything else that you would suggest or recommend generally about HIV prevention or just PrEP availability, accessibility, that new thing or how the community as a whole is our focus on this immediately?

Participant:

A conversation because there's no conversation. The work is being done to certain health workers and bits and pieces, I'm sure. But there needs to be a broader, wider conversation around it. Like, for example, I don't know if PrEP is for straight people. Is it for straight people also that helps them prevent from contracting HIV?

Interviewer:

If it's a straight person with high risk, such as an HIV drug abuser, yes.

Participant:

Okay. So, I just feel like there should be a bigger conversation. There will be some backlash. But here's the thing. If done smartly, it's just a medicine that is preventing HIV. Irrespective of who's consuming it or what person of what sexual preference is consuming it. So that's the conversation that we're missing. And then you see when there's a conversation and conversation lead to curiosity. Curiosity will lead to creating accessibility. It's a chain. It's a snowball effect. And to a certain extent, the snowball effect has been taking shape a little bit, because someone would tell you. But it's word of mouth. And it's very, I don't have the word for it, but it's very tiny. It's like a speck. It needs to be bigger than a speck now. Because you've got your new generation coming, your [the younger generation] who is far more woken and far more accepting of themselves and the surroundings that they're in is more accepting of them. And so, you see, as [our generation], we had no way to go around and how to understand this any better.

But we are figuring it out. But I feel that now, the [young adults] who are coming in, or even the teenagers, are smart. They are bold. They are willing to listen. And it's very important that we start a conversation to keep them safe. Because if you start young, and if you start there, for example, sex education, we don't have sex ed in schools over here. That's so important that if there's a sex education happening around in schools where you just bring up PrEP, where you just let them know, PrEP is available. Or I don't know, of course, I'm 100% sure there's also the same case with pregnancy also, where there's no conversation about it.

This is how it is. If this happens, let's do this. If we follow these guidelines, then we have to sit and let them come to us. If that makes sense. Do you understand? You know what I mean? We haven't done that yet. We haven't made it a free flow of, hey, all right, here we are to answer your questions. Because it's not just a headache where you go and take a Panadol from pharmacy and call it a day, but it's one of those situations where people are scared. They're at their most vulnerable. And especially being Pakistani, you understand how these things can affect the vulnerability and the emotions also. Because we're just talking about our country right now.

We're not talking about the whole world holistically because that's a different setup. So that's very important where we at least have an outlet, to have a conversation and say “We got you. Come, let us help you out. Let us show you what the method is to this madness, how you can be safe and be okay.” That I feel is missing.

Interviewer:

Okay. Absolutely. That's a good suggestion. I think we are done with our questionnaire. If you have any questions for me, I'd be happy to answer.

Participant:

No, I don't. I think all of those things which I felt and saw and heard were very much suppressed and through this interview with you, I think it just came out a lot more, like I got my chance to express my concerns because these are the things that I more often think about. I went I got PrEP and it made me realize of my privilege and thin about those who don’t have that privilege.

I told you a similar incident of my friend having a scare and wanting to get PrEP, that was our privilege to get those two tablets of PrEP which would make us safe for those 72 hours so that window doesn’t pass.

So, there was a little bit of resentment that something as private as [tertiary care hospital] who is getting the material and also the government access, when there is a 3-day weekend, that is when everything is closed and that is when you should be actively available. Because it is during holidays that people have fun. So, when we did it and worked our way around it, we realized it’s our privilege that we worked a way around it and think about those who don’t. Where is the window when one can say for example, they will have their appointment booked for Wednesday but in the meanwhile say “Can I have the tablets so I can be safe.” This is the loophole to be addressed before we even start the conversation. No difficulty in accessing where I just call, go and pick it up.

Interviewer:

Thank you. That makes a lot of sense. Thank you for suggestions and talking to us about your experience generally and your insights and talking about issues, which are so sensitive and personal to you, will be extremely helpful when we analyze. Since you are such a big part of the community and trying to make a difference, if you feel there is any other participant who might be willing to talk to us, we would be super grateful. You could connect them directly with Dr ABC. We are looking at the perspective of the community so can see what exactly the problems are, identify and work on them.

Participant:

Thank you. I really hope we can work on an easier approach which is not just accessible to the privileged men, well-bred ones or the brave ones who know how to fight without fear and do it on their own. Key points are conversation, accessibility and comfort. That’s all we need. Thanks for listening.

Interviewer:

Dr ABC will get back to you with a consent form so you can E sign it or print then sign it, can and then send it back just for the record. It will not contain any identifying information at all. It is a part of the study procedure.

## Interview 5

Interviewer:

Hello, Assalam O Alaikum, can you hear me?

Participant:

Walaikum Assalam, I can hear you, how are you?

Interviewer:

I'm good, thank you for asking, how are you?

Participant:

I'm good.

Interviewer:

Right, is it okay if we start?

Participant:

Yeah, yeah, we can.

Interviewer:

Okay, and would you prefer we conduct this in English or do hybrid?

Participant:

I think hybrid would be alright, if that's okay with you.

Interviewer:

No, of course, that's completely fine. So, I think maybe Dr ABC may have briefed you a little bit about the study. We're trying to understand what difficulties are being faced when people are trying to seek out PrEP, and how much information is out there about it, and what stages the difficulty is being faced, and how people are accepting it either way.

So, I'm just going to ask you a few questions, and this meeting is being recorded. However, the recording will be kept with me and Dr ABC only, and we'll transcribe it into words. In both of these documents, there will be no identifying factor for you, particularly no name, number, anything at all.

And once the study is conducted, sensitive information after a certain period of time also gets destroyed. So, your confidentiality will be prioritized. And if you have any questions at all, during or after, please feel free to ask me. Is that okay?

Participant:

Yeah, thank you so much for sharing all that.

Interviewer:

Of course. So, your participation in the study is completely voluntary. You can choose to withdraw from it at any point in time if you feel you want to do so. So, you can inform the professor, and that will be facilitated for you as well. Okay. So, I'll just get started with the questions, then, if that's okay?

Participant:

Yeah, that's completely all right.

Interviewer:

Okay. So, generally, what do you know about HIV prevention?

Participant:

So, just give me a second. And please repeat your question again.

Interviewer:

Right. So, generally, what general information do you have about HIV prevention? The ways to prevent it, or anything else that you know about it or want to share about it?

Participant:

About HIV prevention, right?

Interviewer:

Yes. Yes.

Participant:

Yeah. So, I mean, first thing first, I know that there are a lot of campaigns that are being run in terms of educating people regarding how they can keep themselves protected from the virus to begin with.

Interviewer:

Right.

Participant:

Other than that there has been a lot of development in the sense that medicines are coming that kind of reduce the probability of somebody getting HIV, given that they are on those medicines. I don't know how true this is, but I also heard that it is also being vaccinated, like it was for COVID. So, that's what I know.

Interviewer:

Okay. And, you know, what steps you are taking for HIV prevention?

Participant:

HIV prevention?

Interviewer:

Yeah. What steps you personally are taking for it?

Participant:

All right. So, there are a few things that I do. First, I use contraceptives to make sure that if I am meeting someone for the first time, just to ensure that I don't get it. Second, I am using PrEP. So, I am on that too. So, that's the second step. Third, I don't know how relevant it is, but I kind of speak with the person who I am sleeping with, because that also helps at times to know their status and how well aware they are of HIV. So, these are three main things that I mostly do.

Interviewer:

Thank you for sharing that. Are we speaking about condoms?

Participant:

Yeah.

Interviewer:

Okay. Great. And how long has it been since you have been on PrEP?

Participant:

It has been for 13 months, to be more precise. I started in [month/year].

Interviewer:

Okay. And generally, what information do you have about PrEP?

Participant:

So, I know that this is a preventive. No, not preventive. How should I put it? So, basically, this medicine helps you not get HIV, in the sense that it is not 100% preventive, but it decreases the probability of you getting the virus. Other than that, I know that there is a very strict schedule that you have to follow, in the sense that if you've started, there is a particular order in which you have to take it, and then you have to abide by that. Other than that, I know that there are certain side effects associated with the medicines, and I do experience some of the side effects whenever I use this medicine.

Interviewer:

Right. Extending from that, what has your general experience been like with PrEP? So, you mentioned side effects, right? Other than that, has the general experience been okay to use?

Participant:

The experience has been alright. I mean, it gives me an upset stomach, but other than that, I don't have any other side effect. I don't know how relevant this answer might be, but…

So, when I tell people that I am on PrEP, they usually are curious about it, and they kind of want to know how to acquire it, and at times they ask me to share it with them, which I don't, because I get limited stock of the medicine myself.

Interviewer:

Have you ever felt the need to stop PrEP in experiences like this, or have you felt that this is too much and maybe you should just stop it? Has that been a factor?

Participant:

No, that's not been the case. Yes, there were times when my stomach got really, really upset. Oh, yeah, I remember. Sometime back, for a month or a month and a half, I had to stop PrEP because my stomach was extremely upset. I don't know what the reasons were. I did get a lot of tests done for my stomach to make sure that there wasn't any virus or something, as in my stomach was really upset. So, for that, I had to stop PrEP in the middle. Because my only visible side effect of PrEP was that my stomach would get upset. So, I had to stop it to make sure that my stomach wouldn't get even more upset.

Interviewer:

And the month you had to stop PrEP, did you feel any difference in your lifestyle generally, or your interaction with your partner? Did it affect you in any way?

Participant:

No, it did not. I mean, during that period, I was only active with one person and the person gets tested once a year. So, I knew that, I mean, it's okay to maybe, you know, stop it for a bit.

Interviewer:

Yeah, okay. And how do you access PrEP? Like, where do you get it from?

Participant:

I get it from, can I mention the name of the hospital?

Interviewer:

Yeah, that's okay.

Participant:

Yeah, I get it from [tertiary care hospital]. Because that's the only place I know where I can get it from. I've heard that there are certain organizations that hand out PrEP, but I don't really trust them as much, and I don't trust them with the data. So, I would rather go to a place where I feel more comfortable, and that is [tertiary care hospital].

Interviewer:

Right. Apart from [tertiary care hospital], do you know any specific hospital or organization? I know you mentioned that you're aware of some, but is there any specific place you're aware of where you can get PrEP apart from [tertiary care hospital]?

Participant:

No, I'm not aware of, like, I don't know the exact names, but I do know that there are certain organizations who do give out PrEP, because whenever I'm speaking with someone regarding PrEP, they do say that they know of some places where they can get it without going to the proper channel, that is [tertiary care hospital]. So, that's how I know. I don't know how credible this information is, but I've gotten this information from a few people, so maybe there is some credibility to this.

Interviewer:

Okay. And how did you find out about [tertiary care hospital] being a point of access for PrEP?

Participant:

So, basically, I was having a discussion with someone, and the person suggested that I visit [tertiary care hospital], because that person also was on PrEP, and the person knew exactly what the procedures are to get it. So, I spoke to that person in detail, and then I followed those steps, and I ended up getting PrEP.

Interviewer:

Okay, nice. May I know how you commute to [tertiary care hospital], and if you have ever been in public transport?

Participant:

I have my own transport. I usually go by a motorbike or a car, depending on whatever is available.

Interviewer:

Okay, and has that been an issue at any point?

Participant:

Not really, but one thing that really bothers me with the entire process is that I have to park my vehicle kind of far, and it takes some time to get to the actual counter where I can get the medicine from. So, that is one of the deterrents, I would say, for me. So, I really have to plan my visit to [tertiary care hospital] when I can go and get the medicines for myself. So, I think that's one of the biggest hurdles that I face.

Interviewer:

Right. What is your understanding of the cost? Like, currently, what is PrEP costing you?

Participant:

PrEP is costing me about, can I mention the specific amount?

Interviewer:

Yeah, of course, that's okay.

Participant:

Yeah, around, I guess, 500 plus minus 100 rupees, because that's the shelving price that I have to pay. That's the price that goes to the pharmacy for storing the medicine in a controlled environment. So, that's what I know of. Other than that, I don't need to pay anything.

Interviewer:

Okay, and has the cost of the process, because I understand there's a certain process you have to go through as well after a certain period of time, along with what you're paying for PrEP. Has the cost ever been an issue?

Participant:

No, not for me.

Interviewer:

Okay, great. Personally, are you satisfied with the way you're accessing PrEP, with the whole process of it?

Participant:

Yes, I'm satisfied with it, because, I mean, I am on-demand PrEP. I don't need to take it every day. So, I mean, my visits to [tertiary care hospital] are not very frequent. So, I don't mind going through the hurdles and going through all the steps that are there in order to acquire the medicine. So, I don't mind, basically.

Interviewer:

Great. You mentioned hurdles here. Do you mind specifying or explaining a little bit about what in the process seems like a hurdle to you?

Participant:

So, for instance, because I'm working a full-time job, right? So, in order to go to the hospital, I need to make sure that my schedule aligns with the doctor's schedule, which is highly unlikely, because I'm usually extremely occupied at work. So, I really have to plan my visit to [tertiary care hospital], when I can exactly go. But once I'm there, then it's okay. And as I said, I need to visit the hospital twice or maximum thrice a year, so it is still manageable. So, that's one of the hurdles that I need to face. Because I really cannot tell anyone why I'm going to the hospital. So, I usually have to make up a story and all that, which I don't feel really comfortable with, because I usually want to be as honest as I can be. But, yeah.

Interviewer:

Well, good point. So, for you, you have to physically go and assess that. Is there any other way that you think it would be better or what, according to you, would be the ideal way to access that, if not this way?

Participant:

The ideal way would be that this medicine is available on any pharmacy of [tertiary care hospital]. So, I mean, visiting the doctor is one thing, but then going to the pharmacy, waiting there in huge lines, waiting for like, you know, 30 minutes and 40 minutes for your turn to get the medicine, that's a big hurdle. But if this medicine is available on all other branches of [tertiary care hospital], then it will make it very easy for me, because there are so many other pharmacies of [tertiary care hospital] where I can go, and usually they don't have a lot of customers at those pharmacies. So, it will decrease the time and make it much easier for me.

Interviewer:

Right, so you only access it through the main [tertiary care hospital] pharmacy, the on-campus one.

Participant:

Yeah, exactly, because that's my understanding that the medicine is available there. And just to share with you, when I met with the doctor last time and they prescribed me the medicine, I could not go to collect it. I just got the receipt and the prescription and came back to my workplace, because I was running late, I had taken a half day, and waiting at the pharmacy would have taken at least another hour. So, that's, again, one of the hurdles that I have to face.

Interviewer:

Did you have to go back to get the medicine then?

Participant:

So, I haven't gone. So, again, I need to plan my visit to [tertiary care hospital].

Interviewer:

Right, so you would have to do an additional visit just to pick up the medicine then?

Participant:

Yeah, exactly.

Interviewer:

Okay, right. So, you told me that you found out about the PrEP through word of mouth with friends. Do you think there's a need, or is there a specific person, professional, you think you should have been able to connect to for information regarding the form?

Participant:

That would have been much easier, because, so, I spoke with the doctor directly, and he really guided me. But, again, when I speak with people about PrEP, and some of the people who are already on PrEP, the way they are taking it is slightly different. So, it is kind of confusing. I mean, if there is maybe like a helpline, or there is some online information available where I can just go and read all the rules and regulations, and the methods, not exactly the methods, but the way to procure this medicine. So, that would be really easy.

Interviewer:

Okay, right. So, do you have any sources of information you turn to right now, if you want to look something up quickly, or if you want to find something about PrEP?

Participant:

No, I don't have any specific resource, but I do know a friend who works in healthcare. I usually contact him for speedy information, but I would say that I'm fortunate to have a friend like that who can guide me, but then there are so many other people who do not have such resources available and such people in their lives, so it might be an issue for them. But for me, I do have someone who I can go to for prompt responses regarding, for example if there's some information that I need regarding PrEP.

Interviewer:

Right. Are you accessing the tablet form of PrEP?

Participant:

Yes, I use the tablet form. I do not know that there are other forms.

Interviewer:

Okay, right. Would you prefer that PrEP be available in any other form at all?

Participant:

I think it's fine. I mean, I don't mind taking tablets. I'm basically good.

Interviewer:

Okay. And do you receive any social support regarding assessing PrEP?

Participant:

Any source of what?

Interviewer:

Social support. Sorry.

Participant:

Social support.

Interviewer:

Right. Any organization, team, group that you look out to for social support?

Participant:

No, no, no, I don't.

Interviewer:

Do you have any concerns regarding your confidentiality or safety with the whole process of accepting PrEP?

Participant:

I'm really sorry. There is some echo. Your voice is not clear. So can you please repeat that?

Interviewer:

Absolutely, I will repeat it. Can you hear me fine right now?

Participant:

I can. Maybe we can switch to Urdu. Maybe that might help.

Interviewer:

Yeah, of course. Sure. Do you have any concerns regarding confidentiality regarding your data or your personal safety when you go to access PrEP?

Participant:

Yes, the issue with confidentiality is that I mean when I go to the pharmacy, the people there know which medicine I'm asking for. So, it's a bit of a stigma that I don't want them to label me. Like, he's someone who's sexually active and he's using PrEP. So that's one thing. The other thing is that I mean whatever the ethics are but my name is written on that receipt. So, whoever is giving me the medicine can get it. Secondly, at home you know my receipts are kept. Anybody can go through my stuff. So, I have to be extremely vigilant and extra careful that my receipts don't end up on someone's hands. So, and if I'm not wrong it's also written on the medicine that HIV Truvada that's the name I think of the medicine.

So, I have to be careful that if it ends up on someone's hands they'll know. I was with one of my friends after picking medicines and we were eating out somewhere. And he opened my book and saw that this was written on it. My friend ended up getting curious and asked why are you taking this and all that. So, I know that these things are necessary as in like if the name of the medicine is written on the medicine, then it must be there. But this becomes an issue that if I have that prescription/medicine then I have to hide it. And other than that, my last concern is that whatever data is there it's being stored somewhere. There is a repository with the government somewhere. Because as far as I know the government is funding it. So, I mean I don't have my utmost trust in the regulatory bodies who are maintaining this data. So, this data can be used against me at some point in time by someone.

Interviewer:

Right. Thank you for sharing that. Did you ever feel that all these concerns because of them, there will come a point where it may make you stop taking PrEP. Because of that you will feel that no, this risk is too much I should stop it.

Participant:

No, not at the moment because all these issues that I have evoked this I am the one who is thinking a little ahead. But there is a possibility that all these things are not there. I mean so many people are using PrEP. Just to answer, you know my concern that someone from the government regulatory body should do it. But why would they do it in the first place and what kind of benefits they can get out of this information. Right. And secondly the other that I have to hide it in house. Because the thing is that I do not want to stop taking PrEP. So, I need to figure out the ways how my hurdles or maybe not hurdles, my insecurities, how to navigate through them basically.

Interviewer:

Right. Has PrEP ever been a point of problem between you and your partner? Have you ever felt disconnected from your partner or they have reacted badly to it?

Participant:

No, no, not at all. And as a matter of fact, my partner encourages the fact that I am being careful and that I am taking preventive measures.

Interviewer:

Right. And your experiences with doctors, healthcare workers who are nurses or doctors or maybe medical students. What are your experiences with them when you are in the clinic accessing PrEP or getting tests done or meeting a doctor?

Participant:

My experience with them is normal. So, when I get the PrEP I usually just speak with the doctor and the interns, I do not interact with them as much. So, it is not a concern. And at pharmacy I do not feel marginalized or feel that maybe I am being judged or anything. Because the lab attendants there or I do not know what you call them but people who are handing out medicine, to me they are also very casual because, I think to them, it is just any medicine maybe.

Interviewer:

Right. Is there a time issue like when you could not get medication on time or like you said there is a specific time when you have to take it before engaging in sexual activity. Has that ever been a problem for you?

Participant:

No, for my better understanding, timing as in like what I am going and collecting or what I am consuming?

Interviewer:

So, both. Has either of these been a problem?

Participant:

I have no issue with what I am consuming. Because I plan in advance as to when I am going to be taking PrEP. I have an issue with when I have to go and pick it up from the pharmacy. The issue is that the pharmacy closes at 8pm and I need to make sure that I am there to pick it up, I have to go till 7pm. If I miss that day then I have to again and plan it for another day. Sometimes, the waiting time is a lot so that also becomes an issue.

Interviewer:

Right. So, you personally get it yourself from [tertiary care hospital]. But in your opinion, who should be responsible for the provision and access of PrEP?

Participant:

Yeah, I think the government should be responsible for that.

Interviewer:

Is there anything that you would recommend or suggest generally regarding HIV prevention and PrEP availability which can benefit you or the community?

Participant:

I would recommend that it should be available to everyone in regular pharmacies. Because currently it is only available in [tertiary care hospital] if we go through [tertiary care hospital]’s channel. Secondly, I think there should be some sort of advertisement or something like to curtail population growth, the government started a lot of campaigns where they would show many ads, advertisements and songs and have very catchy slogans. Exactly, something of that sort that would help. And maybe like more visibility. I know speaking about HIV is a taboo, but maybe more visibility that there are medicines that people can take to basically prevent. Because my understanding of PrEP is that people who have HIV and it is undetected, PrEP is the medicine that is recommended to them. So, basically more visibility of this medicine is going to help people.

Interviewer:

Yeah, so more information that we have an option available.

Participant:

Yeah, but there are two ways, right? So, of one is that the person searches up the information and reaches there or the information is directly coming to that person. Currently, people are looking and searching for themselves and going for PrEP. It should be the other way around as well so the people who do not know about PrEP yet, should get on it as well.

Interviewer:

Right, of course. Okay, so my questions here are done. If you have any questions for me, please let me know. I'll be happy to answer them.

Participant:

Yeah, so will you guys share the findings with the participants?

Interviewer:

So, the findings collectively, we will have a manuscript written. They will be made available for public access once it's approved by the journal. But the findings are collective. It's going to be something like we interviewed X number of participants and this is what we found.

Participant:

Alright, because I know that one of the research ethics is that you show the findings to the participants. So, to ensure that there is nothing manipulated. But then I guess there are so many other ways.

Interviewer:

They will be publicly made available once it's approved by the journal. So medical journals may repost our findings, right? So, they have to be approved by them before they're with the general public. And once it's available or once it's approved, it gets published online and you can easily access them.

Participant:

Okay, perfect.

Interviewer:

Okay, are there any other questions I can answer for you?

Participant:

No, no, that's it.

Interviewer:

Okay, thank you so much for your time. And if you have any people in the community, friends or anybody else who you think would be interested in talking to us or being part of the study, you can let them know about it. They can contact Dr ABC if they are interested.

Participant:

But do they need to be on PrEP?

Interviewer:

Yes, they will have to be on PrEP. Either they will need to be on PrEP or they will be interested in looking out for PrEP.

Participant:

Alright.

Interviewer:

Okay, thank you so much.

Participant:

You're welcome. Allah Hafiz.

## Interview 6

Interviewer:

Assalam O Alaikum, can you hear us?

Participant:

Hello, how do you read me?

Interviewer:

Your voice is very muffled.

Participant:

Is it better now?

Interviewer:

Yes, yes, it's much better. So, Assalam O Alaikum, thank you so much for joining us.

Participant:

I'm sorry, I'm a little late.

Interviewer:

No, no, it's completely fine. It's good that you gave us time. Okay, you must have read the consent, right? Dr ABC has discussed with you about the study.

Participant:

Yes, yes.

Interviewer:

So, I will take a verbal consent from you that whatever information you give will be anonymous. And it's a completely voluntary participation. And if you feel that you have to withdraw, you can always do so. So, do you allow us to start the interview?

Participant:

Yes, yes, I'm good with it. Even if it's not anonymous, as I told Dr ABC, it's good to spread the word around for the cause. So, yes.

Interviewer:

So, are you comfortable with Urdu completely or English completely?

Participant:

I think a mix of both. Or do you want to keep it on one medium? What do you guys want to do?

Interviewer:

Okay, so I'll ask. So, I'll ask the questions in English. And if you feel that you need to rephrase anywhere, make sure to tell me, so I can translate it to you in Urdu. Okay, okay.

Participant:

Okay. Good.

Interviewer:

So, thank you so much for becoming the participant, voluntary participant of this study, exploring the barriers, willingness, and preferred ways of using PrEP among MSM population in Karachi, Pakistan. So, I would like to start my interview with the first question, which states, what do you know about HIV prevention?

Participant:

Okay. So, HIV is a virus. To prevent it, during intercourse, we need to have a prevention, which can be used in the form of condoms. So, knowing the status of the person, first, is he positive or negative, which is very difficult, because people don't really disclose it. And regardless, even if we do disclose it, we are not sure whether the report is valid or not, because there are so many softwares around the market, which can be negative or positive. I discussed this with Dr ABC, we have a case here. So, yes, preventing it with the support of condoms, and knowing the status of the person who we are seeing or meeting with. And prominently the usage of PrEP.

Interviewer:

So, do you think PrEP is the only way of prevention?

Participant:

It is not the only way, but it aids in the process, I think. Because even in this, 100% efficacy is not there. Still, there is 99.7%, but usually it covers most of the part. But then there are again some cons of taking PrEP, which we will discuss further.

Interviewer:

Okay, you tell me, according to you, what steps do you take specifically for HIV prevention?

Participant:

So, I do get myself tested twice yearly, biannually. There are 2-3 tests, which I go through on a regular check, like HIV, VDRL, RPR, and TPHA. I think these 3 tests cover the most part of my sexuality. And together with taking PrEP, knowing the status first and then taking PrEP, and still making sure that the person I am meeting with should be on a safer side as well. Which is a little difficult here. But now, after taking PrEP, I don't prefer using condoms. Which was a practice earlier, but now I don't really feel like doing it. Which I think should be taken care of.

Interviewer:

Okay, what are your concerns regarding confidentiality and safety while accessing PrEP?

Participant:

Okay, I understood safety and confidentiality.

Interviewer:

Because we are often scared that people might know that we are taking PrEP, or we are people living with HIV. Exactly. We have these concerns.

Participant:

In the [a large city in the country] community, there is a myth that people who are taking PrEP are considered positive.

That's why they are taking it. This is a passive approach towards taking PrEP. It's either positive or dubious. So rather than appreciating that someone is taking PrEP, they put you in doubt. He must be or he might be positive. So, this is also a concern that I try to make people aware. And what was your second question? Safety?

Interviewer:

Yes. Safety regarding taking PrEP.

Participant:

PrEP, I've been in this program for more than two years now. I do it pre and post regimen. Because I started taking it daily as well, but I felt that it bothers the body systems. It varies from brand to brand. Now that this is the current brand that I'm using. It's a heavy drug. We do workout as well and it has side effects on lethargy as well. So, I think, I take it on pre and post. And since I'm not just sexually active as well so it's easily manageable for me to do pre and post rather than taking it daily and just because of the symptoms I feel aggressive.

Interviewer:

Have you ever felt stigmatized while accessing PrEP in your own experience?

Participant:

Yes, that's what I was trying to say. If you disclose it to someone that you are using or taking PrEP, they just stigmatize you that he might be positive. This is why he's taking PrEP. And that he's taking PrEP only when, he's positive. So, we have a lack of awareness. I think we should go to the platforms and forums and talk about it. There should be an awareness week or awareness sessions so that people know that PrEP is a prophylaxis prevention. It doesn't mean that the person is himself positive.

Interviewer:

So, now it's a very open-ended and general question. You can share any of your experiences. So, if you use PrEP, what are your experiences with using PrEP? You can share any of your experiences.

Participant:

I think I've pre-answered many questions. But I think the usage of PrEP is that I feel it's a heavy drug as well. I know it's important. I know it's needed. But it's a little tough to intake it regularly. And since I do it like pre and post, it's an unplanned activity as well. So, in that situation, it gets tricky and tough because you have to take it 2 hours before at least to get it into your system. So, sometimes that time slot gets a little disturbed and missed. So, this is a little thing that I find it a little difficult to maneuver on pre and post. And the second thing is lethargy and the body feels weak if you take it daily.

And I've noticed a difference between brand 2 and brand 3. The current brand I'm using, I don't know what it's called. The previous one was Mylan. That was better. But this is a heavier one. I take it for 2-3 days. So, I feel it in my body. My body is breaking. There is little adaptability to this drug. It's not just friendly. And I remember Dr ABC said that if you're taking it on a daily basis, then you have to go for your creatinine test as well. This is a little heavy. And maybe the supply which we are getting here in Pakistan, there are other friends of mine as well abroad and their drug is not that heavy, so maybe this is the reason. Truvada, I guess, its composition is different. I'm not sure this is all your domain.

Interviewer:

So, with all these experiences that you are sharing, have you felt that you should stop or not use PrEP?

Participant:

No, no, no. This is something which I can't stop now. Since I've started and enrolled in this program and since I know the importance of this drug. So, I can't stop it. In fact, I try to make people aware around me. I try to get them on the program as well and on the PrEP as well. So, I can't do that.

Interviewer:

So, how long you have used PrEP and where do you get it from?

Participant:

I think it's been more than two years. You can cross check it with your system. Since I started seeing Dr ABC, I started with the program. So, more than two years, I guess. It was [year], so more than two years, more than three years now. But I do it on pre and post. So, my drug has been going on for a long time. And I get it from [tertiary care hospital] as well.

Interviewer:

Okay, so there are two components which might look very simple but they are very important in terms of accessing PrEP. One is commuting, which means transportation to the pharmacy, hospital, etc. and accessing PrEP. And the other one is cost, which means buying it. So, do you have any experiences related to that? Or do you think what is the cost? How is it in commuting?

Participant:

Since I get it from [tertiary care hospital], the cost is higher as compared to the other NGOs. Seeing the doctor and then getting it prescribed and then going to the pharmacy. Then there are different charges. So, I think the process can be smoothened and since other NGOs are competitive in the market, they do it from testing to handling PrEP. It all happens in one day or within a few hours. Testing is done immediately. They go and consult a doctor and then they give you the PrEP. So, it is a very precise process and there is no cost at all. They just have to go to the NGO. I think [public hospital] is also giving. Sorry, [public hospital]. So, there is another friend who got it from there and it was done in 3 hours. Here, I think they do the testing first. The charges of [tertiary care hospital] are such that HIV test is around more than 3000. So, seeing a doctor and then if they take the charges under [the national HIV program] from pharmacy, then you get a good charge of around 7000-8000. More than 7000-8000, within 10,000. So, seeing a doctor and then getting it prescribed, testing is separate, this is a bit of a drawback. For this reason, people who I do refer as well to [tertiary care hospital], they avoid it. They say after hearing the name that it is a bit expensive.

Also, actually this might cover in the next question.

Interviewer:

No no, you can always continue. No problem.

Participant:

Okay. So, I was saying that people who prefer discreetness because going to NGOs can stigmatize you a bit. Because the people who are operating these NGOs are basically from this community as well. Because people are like that. And so, going out there and rather than being more professional, it becomes a bit of a personal intervention in your personal space because people might know you and they might relate to other people. Like, this person came to me today and got tested. And what if somebody tests positive? Then it's a grapevine thing in the community. Because the community is a very small place. And things don't get discussed here. So, you get very stigmatized there.

In [tertiary care hospital] it gets very professionalized, the whole process.

Another thing that I was concerned about. Since I'm opening it up, I'll talk about these things openly. This, after COVID, I was talking to Dr ABC as well, I predicted that HIV cases will increase due to the usage of few drugs. I think we'll talk about this later. That's all. Please remind me.

Interview:

How can I remind you? Give me a hint about what you were talking about.

Participant:

We'll talk about drugs later if you have any questions.

Interview:

Okay. And we'll definitely take this over. You have shared details about costing. But would you like to talk about commuting? How is it in terms of accessing, commuting, transportation? Is it far?

Participant:

Yes, it's far. Even I was running out of my stack. So, I was thinking and people don't take the bus from [the area near the tertiary care hospital]. So, it's a different cost coming all the way to [tertiary care hospital]. It's not a one-time thing. You need to see a doctor and then get it recommended. Then you need to check if PrEP is available or not. Last time, it was not available so, I chased them. I called and confirmed from [the hospital drug information service]. So, the process can be smoothed. One recommendation can be that if you guys can make it kind of a package thing so, the process can be smoothed and the waiting time or down time can be reduced. I checked the doctor; the testing is done and PrEP is received. So, it should be a kind of a package so it will be easy for people.

Interview:

You mentioned about the NGOs and other things. Do you receive any social support regarding accessing PrEP from NGOs, family, friends apart from [tertiary care hospital] or the places you mentioned.

Participant:

I was thinking in the mind that I should go to an NGO because the [tertiary care hospital] charges were too high. This is what I discussed with Dr ABC as well, that the charges started from 1700 and it went up to 1900. Now, it's nearer to 4000. I don't know about the current. Maybe it's more than 4000. So, I myself was thinking of going to an NGO and continue it from there then Dr ABC said that you have to see me only once a year. Then, I did as once a year is fine but, the cost really hits the people. The cost in the whole process.

Interview:

Okay. So, this whole cycle of accessing PrEP, are you satisfied with the way you are accessing PrEP?

Participant:

I told you that the process can be smoother with the [tertiary care hospital].

Interview:

Would you like to talk more about this in detail? Can you talk about it in detail?

Participant:

The process, I told it in detail earlier. But I can repeat it. The process can be smoothed in acquiring the PrEPs. First of all, offering prospective patients to have a package in which all these things are covered such as testing, seeing a doctor as well as getting PrEP. So, this process can be subsidized a little and converted to one day. Like other NGOs which take 3-4 hours. Testing, seeing doctor and giving PrEP is done in 3 hours. Guidance is also given and they all get it done free of cost. Whereas in [tertiary care hospital] we need to pay under Rs. 10,000 for the whole thing. Seeing a doctor, getting it from the pharmacy as well as testing which is expensive.

If they can offer something in the package, something subsidized. Because already the drugs are on [the national HIV program]. It is not on [tertiary care hospital]'s cost. So, seeing it, this is a public health concern and since after COVID, this has multiplied due to the intervention of drugs in the process. So, this is spreading.

Interview:

I would like to rephrase the question because what you are saying I think are recommendations. It is a good thing and we will note it there. But this question is specific to you only, this is based on your experience. The way you are accessing PrEP, do you think you are satisfied or not?

Participant:

I am satisfied. But I will still go with the same notion for myself as well as I told you.

Interview:

Okay. So, I think. Multiple things and multiple questions you have covered together. We will break it down. Okay, you mostly talk about [tertiary care hospital] experience. Do you think there are any specific experiences with healthcare personnel while accessing PrEP. For example, nurse, physician, pharmacist, resident.

Any relevant experiences. With healthcare providers while you are accessing PrEP.

Participant:

Yes. I face some trouble in pharmacy. Because they think it is used for only patients who are positive. They don't know it is for prevention. So, they need some guidelines because I want to make them understand that I don't take it daily. Like Dr ABC gave me which was for 3 months and the stock was expiring. So, I told him to give it to me for 1 month and carry forward the rest. So, he said you have to take it in 3 months and after 3 months the stock will expire. So, they have to understand as they think that because the doctor said it is for daily use. They don't know it can be used pre and post as well. My bottle can be used for 3 months or 4 months which is usually 2 in my case because I take it pre and post. So, if a doctor has prescribed me for 3 months it can be used for 1 year. But in pharmacy, they say you have to take it in 3 months, after 3 months your prescription will not be valid because the doctor said it is for daily use. I think all parties have to be on board in [tertiary care hospital] to know this is a specialized drug like cancer. It has protocols and variability in it.

Interviewer:

Of all the experiences you have discussed so far, do you think there should be an ideal way to access PrEP? For example, it would have been better if it was like this.

Participant:

The ideal way is that you should see a doctor, they should prescribe it, you get it from pharmacy.

All in minimum cost just like NGOs. This is why people prefer going there, to switch there because of the cost.

Interview:

So, you told finance and commuting. Do you have any limitation related to time? Do you think it takes a lot of time?

Participant:

Yes, since it is to be prescribed first by doctor. Patient enrollment is so high that I have waited for 3 hours as well to get prescription only. It is very time-consuming. And there was another friend whom I recommended, who came all the way from [abroad] and got an appointment as well. The waiting time was too much so he just walked away. This should be approached viewing it as a specialized program or specific days for PrEP patients but this might breach confidentiality to each other as well.

Interviewer:

In what form do you feel that PrEP should be available? It is in tablet form; do you think it is fine or there should be injections instead? Or any other form?

Participant:

No, I think oral tablets are better. I have one concern, is it the same formula which people are worldwide getting?

Interviewer:

I think Dr ABC would be the better person to respond to this question. We just want to know that the people whom we are interviewing, do they have any thoughts that this tablet is not right, there could have been an injection?

Participant:

This is why I was asking because my friends who are using it globally, their drug is not that heavy. Because they do take it daily and they don't feel the side effects because I take it 3 or 4 days, the current batch prominently and I feel the side effects. The side effects are an element which sometimes I feel whether I should take it.

Interviewer:

I think we will convey your question to Dr ABC so definitely he can respond to you so that you don't miss out on this. Tell me if there are any specific people whom you feel you should contact or you contact in terms of getting any information or services regarding PrEP?

Participant:

I think I get first-hand knowledge from Dr ABC. I disseminate this to other friends and people I have known or people who seek advice from me. Your end is the first-hand information which I get.

Interviewer:

One very important question is, what are your feelings and perceptions about connecting with your sexual partners while you are using PrEP?

Participant:

Sorry, say again, I just missed it, the first part.

Interviewer:

What are your feelings and perceptions about connecting with your sexual partners while you are using PrEP?

Participant:

It's the same, I think I feel more confident, I feel more secure, I feel more covered. So, this is a pro which I would count, having PrEP on and eating something.

Interviewer:

Would you like to unpack this word “covered”?

Participant:

Covered as in the drug is in my system and it's preventing me, it's protecting me from the possible chances of infection with the virus which the other person has. But then again, the con side is that I do now take care of having no condom while under PrEP. So, this is one thing which I feel is leveraged, which I can skip, which I think is not the cool part of it.

Interviewer:

I think we are almost wrapping it up with one or two more questions. You have given a lot of recommendations. I just would like to know; do you have any other recommendations or suggestions regarding HIV prevention and PrEP availability or PrEP accessibility?

Participant:

My number one recommendation is to make the process smoother, swifter. From the [tertiary care hospital] and pharmacy, consultation clinics, they all should be on board in the process. Because people don't think this is important. And then when they see this process, they realize it's such a long process and costly process. Showing it to the doctor, its charges, testing charges and then getting it from the pharmacy. Then those who have to take it, skip it. So, I think you can make the process a little cheaper. Or you can offer a package where all three or four things can be offered at the same time. Or a specific day where you can only address these people. Or something, any mechanics related.

Secondly, I think awareness is very important. Being one of the leading hospitals like [tertiary care hospital], like we celebrate awareness days if there is an awareness day for this, you can organize one in an auditorium. Or a public service message like what's on TV, radio or on the mass media to get people aware of the thing. HIV, its reality, its spread on public forums or the events that are happening in [a large city in the country]. I was talking to the doctor yesterday. Because the community people are more interested in arts, channelizing it like this way for awareness can be done. Because the straight people who are not from MSM, they also need awareness. Because it gives a mass awareness if there is an art event in [an arts venue], or [an arts venue], or anywhere. So, in the end, you can go to the representative and give a 5–7-minute awareness speech.

So, this would all contribute towards the cause. And the other thing that I talked to you about. After COVID, I was predicting that the spread would increase and it did. The reason for that is that during the COVID, the government shut down everything such as activities, sports, schools. I predicted earlier that there would be a chunk of teens like grown-up and maturing teens who would come into this market due to the usage of drugs. Now, there are so many drugs in the market after the COVID which people use immensely. One is Methamphetamine which people call “ice”. Now, this has made it the worst scene in the masses. People are having orgies. Because this drug has such a tricky mechanics. It makes extroverts super confident and people avoid all the preventions and they do go super vulnerable. There are groups and orgies happening. 5, 6, 7 people. You don't know if somebody is positive. If it was not under drug, people would have used it for prevention. But this stimulant is so strong that people do let it go no matter how many preventions there are. Now, there was just one case. It just happened earlier in [a month]. There was just one kind of group happening somewhere in the city and one guy came up with these remote HIV kits and he tested. He said, I will join only if everybody gets themselves tested. Everybody did, out of those 6 to 7 people, 4 came positive. I don't know the efficacy of those kits. But still, this is an alarming situation. All were under the influence of Methamphetamine. Because you go as superman. You can do anything and everything. There isn't any inhibition. So, I think the youngsters don't take it this way. They don't know anything about the handling of the drugs or anything. Is it causing something to the brain and other things or the spread of HIV? They really don't know anything about it. So, this is what is happening.

Interviewer:

Thank you so much for sharing this in so much detail. I think it is very helpful and we should dig out things from this.

I just want to conclude with the question, according to you, who is accountable for provision or access to PrEP?

Participant:

I think the government obviously. I always have the confusion, is it for the PrEP prophylaxis or for HIV prevention.

Interviewer:

HIV treatment is ART. We are talking about PrEP.

Participant:

This should be responsibility of the government and then the respective departments or hospitals in healthcare. Be it [tertiary care hospital]. I think they are doing a good job but mass awareness is necessary because people don’t know whether this thing is available or not. I am in this program for 2 years and people don’t know we have PrEP available in Pakistan. It is a mutual responsibility even for other people who are using it should make other people aware of it.

Interviewer:

Most of the information that you have shared, you also mentioned about [tertiary care hospital]. Do you have anything to share about PrEP generally which you think was missed out?

Participant:

I think I have said it all. I hope it helps for betterment.

Interviewer:

Thank you so much for your time. I think you have already signed the consent form, right?

Participant:

Yes

Interviewer:

Dr ABC will be the point person for this study so if you have any questions, you can always contact Dr ABC. I am Sarmad, me and Preet are from Dr ABC’s team for this specific study. Take care. Allah Hafiz.

Participant:

Allah Hafiz.
